# Genome-wide screening identifies Polycomb repressive complex 1.3 as an essential regulator of human naïve pluripotent cell reprogramming

**DOI:** 10.1126/sciadv.abk0013

**Published:** 2022-03-25

**Authors:** Amanda J. Collier, Adam Bendall, Charlene Fabian, Andrew A. Malcolm, Katarzyna Tilgner, Claudia I. Semprich, Katarzyna Wojdyla, Paola Serena Nisi, Kamal Kishore, Valar Nila Roamio Franklin, Bahar Mirshekar-Syahkal, Clive D’Santos, Kathrin Plath, Kosuke Yusa, Peter J. Rugg-Gunn

**Affiliations:** 1 Epigenetics Programme, Babraham Institute, Cambridge, UK.; 2 Department of Biological Chemistry, David Geffen School of Medicine, UCLA, Los Angeles, CA, USA.; 3 Wellcome–MRC Cambridge Stem Cell Institute, Jeffrey Cheah Biomedical Centre, University of Cambridge, Cambridge, UK.; 4 Stem Cell Genetics, Wellcome Sanger Institute, Hinxton, Cambridge, UK.; 5 Cancer Research UK Cambridge Institute, University of Cambridge, Cambridge, UK.; 6 Stem Cell Genetics, Institute for Frontier Life and Medical Sciences, Kyoto University, Kyoto, Japan.

## Abstract

Uncovering the mechanisms that establish naïve pluripotency in humans is crucial for the future applications of pluripotent stem cells including the production of human blastoids. However, the regulatory pathways that control the establishment of naïve pluripotency by reprogramming are largely unknown. Here, we use genome-wide screening to identify essential regulators as well as major impediments of human primed to naïve pluripotent stem cell reprogramming. We discover that factors essential for cell state change do not typically undergo changes at the level of gene expression but rather are repurposed with new functions. Mechanistically, we establish that the variant Polycomb complex PRC1.3 and PRDM14 jointly repress developmental and gene regulatory factors to ensure naïve cell reprogramming. In addition, small-molecule inhibitors of reprogramming impediments improve naïve cell reprogramming beyond current methods. Collectively, this work defines the principles controlling the establishment of human naïve pluripotency and also provides new insights into mechanisms that destabilize and reconfigure cell identity during cell state transitions.

## Teaser

Genome-wide functional screening reveals new mechanistic principles that establish human naïve pluripotency.

## INTRODUCTION

Pluripotency, the ability of individual cells to give rise to all the tissue lineages of a mature organism, is a fundamental process that we have yet to understand fully. In human development, pluripotency emerges in the unspecialized epiblast cells of preimplantation embryos and lasts for 2 weeks until the postimplantation embryo gastrulates and lineages are specified (*1*). During this period, pluripotent cells isolated from embryos give rise to unspecialized human pluripotent stem cells (PSCs) in the culture dish that retain their developmental potential and characteristics (*2*–*5*). Pluripotency is also acquired when somatic cells are reprogrammed to become induced PSCs (iPSCs), resulting in a cell type that is largely indistinguishable from embryo-derived PSCs (*6*, *7*).

PSCs exist in two main states that are termed naïve and primed (*8*, *9*). Both cell states can self-renew and undergo multilineage differentiation but are functionally and molecularly distinct. Naïve PSCs largely recapitulate the transcriptome, epigenome, and differentiation potential of preimplantation embryos, and primed PSCs are similar to early postimplantation embryos (*10*–*16*). This developmental identity uniquely endows naïve PSCs with sought-after properties, including the ability to generate extraembryonic cells and entire blastocyst-like structures (*17*–*24*) and for providing a model to study early developmental events, such as X chromosome inactivation (*11*, *12*). Naïve PSCs can be obtained directly from human preimplantation embryos, but more commonly, these cells are generated by reprogramming primed PSCs to a naïve state by exposing them to conditions that induce their cell state conversion (*25*–*31*). However, as with most reprogramming systems, the efficiency of reprogramming to a naïve state is low and produces a high level of cell heterogeneity (*28*, *29*, *32*). Furthermore, the vast majority of induced somatic cell reprogramming experiments generate primed PSCs and not naïve PSCs; as a result, we know very little about the reprogramming of human cells to naïve pluripotency. Recent studies have described transcriptional changes that occur in cells during naïve reprogramming (*29*, *32*); however, we do not know which factors and pathways are involved or are required for this process. Thus, there is a fundamental gap in understanding the mechanisms that control the entry of human cells into naïve pluripotency, thereby hindering our knowledge of early human development, limiting improvements to reprogramming protocols, and preventing the full potential of these cells from being achieved.

## RESULTS

### Defining the essential regulators of human naïve cell reprogramming

**Fig. 1.**
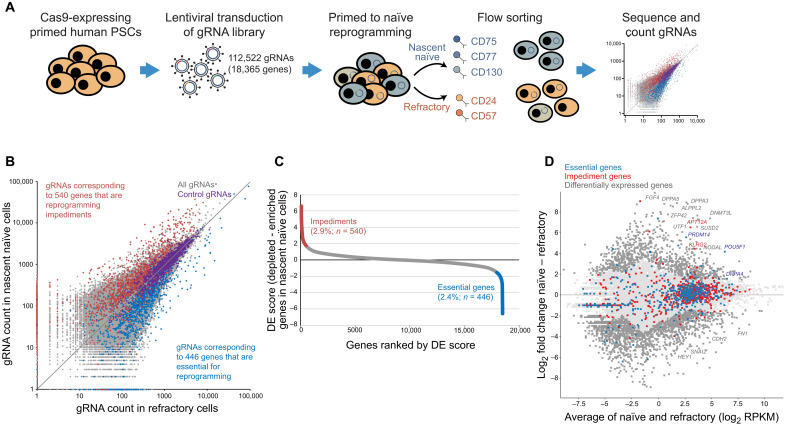
CRISPR-Cas9 knockout screen defines new regulators of naïve PSC reprogramming. (**A**) Screening strategy to identify regulators of naïve PSC reprogramming. Cas9-expressing primed PSCs were transduced with a gRNA library targeting the exons of 18,365 genes. The library also contained 1004 gRNAs targeting negative control regions. Puromycin-selected transduced cells were reprogrammed to naïve pluripotency under 5i/L/A conditions for 10 days. The cell population was cell-sorted using a panel of cell surface markers into nascent naïve cells (successfully reprogrammed) and refractory cells (not reprogrammed). High-throughput sequencing counted gRNAs in the sorted cell populations. (**B**) Scatterplot shows the counts of each gRNA in the nascent naïve and refractory cells. Each gene is targeted by an average of six gRNAs. Red, gRNAs that target impediment genes; blue, gRNAs that target essential genes; purple, negative control gRNAs; gray, all other sgRNAs. (**C**) Ranked differential enrichment (DE) plots by comparing nascent naïve with refractory populations. Numbers of identified impediment and essential genes are shown (applying a cutoff of *P* < 0.02, permutation test) together with the percent out of all genes targeted. (**D**) MA plot shows that the majority of essential and impediment genes are expressed at similar levels in nascent naïve and refractory cells. Transcriptional data are shown for each gene (represented by a dot), comparing nascent naïve and refractory cell populations; dark gray, differentially expressed; light gray, all other genes. Essential and impediment genes are colored in blue and red, respectively.

We set out to define the genes that regulate the reprogramming of primed PSCs into a naïve state using a genome-wide CRISPR-Cas9–based screen. We first integrated the Cas9 coding sequence under the control of a CAG promoter into the safe harbor *AAVS1* locus in primed PSCs and confirmed that this modified cell line could reprogram to a naïve state with the expected proportion of naïve cells within the population (fig. S1, A and B). Cas9-expressing primed PSCs were then transduced with an optimized human v3 CRISPR-based loss-of-function mutant library at a multiplicity of infection (MOI) of 0.3 and a library representation of >100 cells infected per single-guide RNA (sgRNA). The library consisted of 112,522 sgRNAs targeting 18,365 genes and 1004 gRNAs targeting negative control regions (data S1). The sgRNA plasmids also contained puromycin resistance and blue fluorescent protein (BFP) markers (fig. S1A). After 3 days of puromycin selection, >95% of cells expressed sgRNA plasmids, as revealed by flow cytometry analysis of BFP signal (fig. S1C). Transduced cells were reprogrammed using 5i/L/A conditions (*26*), and on day 10, a panel of cell surface markers (*32*) was used to flow-sort two populations: (i) nascent naïve PSCs (i.e., successfully reprogrammed) and (ii) refractory cells negative for naïve markers (i.e., not reprogrammed cells) (Fig. 1A and fig. S2A). Prior colony-forming assays and molecular characterization showed that the reprogrammed population at this stage, although comprising <5% of the cell population, contained all of the cells capable of generating naïve cultures (fig. S2, B and C) (*32*). Genomic DNA was extracted from the two flow-sorted cell populations, and the abundance of each gRNA was measured by high-throughput sequencing. RNA sequencing (RNA-seq) libraries were also prepared from the same samples, and as expected, the transcriptional profiles of the two cell populations showed a strong correlation with previous naïve cell reprogramming experiments (*R* > 0.95; fig. S2D) (*32*).

Comparing gRNA counts between the two isolated cell populations using the MAGeCK algorithm (*33*), which takes into account the multiple sgRNAs per gene, identified gRNAs that target 446 genes (2.4% of all genes targeted) that were significantly underrepresented in the nascent naïve cell population; these genes are therefore essential for reprogramming (*P* < 0.02, permutation test; Fig. 1, B and C, and data S2). Conversely, we identified a similar number of genes (*n* = 540; 2.9%) whose gRNA counts were significantly overrepresented in the nascent naïve PSC population, which correspond to genes that impede reprogramming and whose targeted deletion led to enhanced reprogramming (*P* < 0.02, permutation test; Fig. 1, B and C; and fig. S3, A and B; and data S2). Examining the distribution across cellular compartments revealed that, compared to the background set of genes, essential and impediment genes encode proteins that are strongly enriched for factors localized in the nucleus (*P* < 5 × 10^−6^, Fisher’s exact test; fig. S3, C and D). Only a minority of the essential and impediment genes identified in the screen changed their expression levels between primed PSCs and nascent naïve cells (2.3%; fig. S3, E to G) or between reprogrammed and refractory cells (2.2%; Fig. 1D). This finding shows that the induction of new genes is not typically required for primed to naïve reprogramming but instead that existing factors are repurposed to induce a cell state change.

### Essential regulators of naïve cell reprogramming are distinct from genes required for primed cell reprogramming or proliferation

**Fig. 2.**
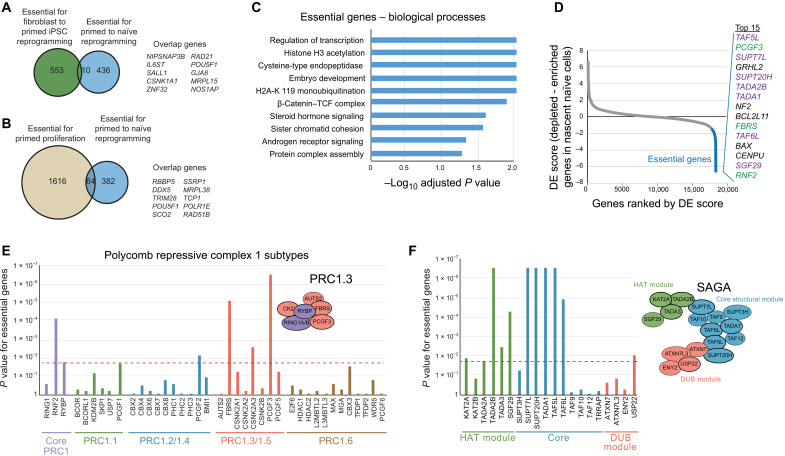
Analysis of essential genes identifies new roles for PRC1.3 and SAGA complexes in naïve PSC reprogramming. (**A** and **B**) Venn diagrams show the overlap between genes that are essential for naïve PSC reprogramming and genes that are essential for (A) fibroblast to primed iPSC reprogramming (*34*) and (B) primed PSC proliferation. (**C**) Charts show the adjusted *P* value for essential genes in the biological processes gene ontology (GO) category (Fisher’s exact test). (**D**) Ranked DE plot by comparing nascent naïve with refractory populations. Essential genes are highlighted. The top 15 ranked essential genes are shown; SAGA components, purple; PRC1.3 components, green. (**E** and **F**) Charts show the *P* values of (E) PRC1 members and (F) SAGA complex members, as a measure of their depletion in the nascent naïve cell population following the CRISPR-Cas9 screen. The red line indicates *P* = 0.02 as a cutoff (permutation test). Schematics of the complexes are also shown; bold lines indicate components classified as an essential gene.

We next examined the genes that were identified as being essential for primed to naïve PSC reprogramming. There was only a small overlap (~2%) between our list of essential genes and the genes that are required to reprogram human fibroblasts into primed-state induced pluripotent stem cells (iPSCs) (Fig. 2A) (*34*). Genes that are common to both studies include *SALL1*, *ZNF32*, *RAD21*, and *POU5F1* (also known as *OCT4*). Examining the differences, we found that many of the genes that are essential for fibroblast to primed iPSC reprogramming are associated with pathways that mediate cell adhesion and mesenchymal-to-epithelial transition, which are processes that are less relevant in primed to naïve PSC reprogramming because primed pluripotent cells are epithelial. Overall, these results suggest that the factors required for entry into the naïve pluripotent state are largely different from those required for converting somatic cells to primed pluripotency.

In addition, to stringently determine the effects of essential genes on naïve reprogramming rather than on primed PSCs, we integrated the results from a second genetic screen in the same primed PSC line, which identified genes required to maintain the proliferation of primed PSCs. We identified 64 essential genes in our reprogramming screen that overlapped with the list of genes that are required for primed PSCs (*P* < 0.02 in primed PSC screen, permutation test; Fig. 2B). Because these common genes might have roles in sustaining pluripotency in multiple states, we prioritized in follow-up experiments the 382 genes that are implicated in naïve reprogramming (data S2). Together, these findings have identified the factors that regulate naïve PSC reprogramming and establish that essential regulators of naïve cell reprogramming are mostly distinct from those genes that are required for primed PSC reprogramming or proliferation.

### Signaling, transcription, and chromatin regulators including Polycomb repressive complex 1.3

The genes essential for naïve cell reprogramming are strongly enriched for functions associated with transcriptional regulators and in chromatin modifying pathways (Fig. 2C and fig. S3, H to J). Visualizing the essential factors based on their known interactions revealed that many, sometimes all, proteins within a particular complex were identified, thereby implicating not only individual genes but also whole complexes that are required for naïve PSC reprogramming (fig. S4A). Most of the identified genes and complexes have no prior connections to naïve reprogramming or human pluripotency.

In particular, the top ranked hits included multiple members of the Polycomb repressive complex 1.3 (PRC1.3) and the SAGA (Spt-Ada-Gcn5 acetyltransferase) complex (Fig. 2, D to F). PRC1 complexes typically repress transcription, acting through the mono-ubiquitylation of histone H2A lysine 119 (H2AK119ub1), the recruitment of PRC2, and chromatin compaction (*35*). PRC1 consists of the ubiquitin ligases RING1A/B and one of six homologs of the Polycomb group RING finger (PCGF1 to PCGF6) proteins that define distinct subunit assemblies. Canonical PRC1 contains a Chromobox (CBX) protein that binds H3K27me3, whereas variant PRC1 lacks CBX and contains RING1 and YY1 binding protein (RYBP)/YY1 associated factor (YAF) and subunit-specific auxiliary factors. In our screen for factors essential for naïve PSC reprogramming, we identified *RING1B*, *PCGF3*, *FBRS*, *RYBP*, and several *CK2* genes within the list of significant hits, and these genes comprise nearly all of the known components of PRC1.3 (Fig. 2E). *PCGF3*’s close homolog, *PCGF5* (a component of PRC1.5), had only a minor impact on naïve PSC reprogramming in our screen, presumably because *PCGF5* expression is low in PSCs. The identification of PRC1.3 was specific: Out of the other PRC1 subtype components, only *PCGF1* and *PCGF2* were also essentially required for naïve PSC reprogramming; however, no other PRC1.1-, PRC1.2-, or PRC1.4-specific components were within the set of essential genes (Fig. 2E).

Another prominent complex identified in the screen as being required for naïve reprogramming was the multiprotein SAGA transcriptional coactivator complex. Of 18 core SAGA components, 7 ranked within the top 15 hits in our screen (Fig. 2D) and 11 were within the list of genes essential for reprogramming (Fig. 2F). Most of these genes are not required for generating nonreprogrammed, refractory cells (data S1) or for primed PSC proliferation, indicating that SAGA function is needed specifically for naïve reprogramming. None of the SAGA components significantly changed their expression levels during reprogramming (fig. S4B). SAGA functions through multiple catalytic activities, including histone acetyltransferase (HAT) and deubiquitylation (DUB) (*36*). Our screen results point to an essential role for the HAT GCN5 (KAT2A) and other members of the HAT module, with a less-prominent role for the DUB module, with only one of the four components required (Fig. 2F). Other large regulatory complexes with components in the cohort of essential factors included the Mediator complex (fig. S4C). Additional regulators with core cellular functions were also identified (fig. S4A), which, as expected, were also required for primed PSC proliferation (Fig. 2B).

Our genetic screen also identified signaling components and transcription factors that were essential for naïve PSC reprogramming (fig. S4, D and E). One example is for components involved in the assembly of the WNT/β-catenin–T cell factor (TCF) complex (*q* = 0.01, Fisher’s exact test with Benjamini-Hochberg correction; Fig. 2C and fig. S4E). All components are highly expressed in the pluripotent epiblast cells of the preimplantation human embryo, raising the possibility that similar signaling events could also be present during embryo development (fig. S4F). The identified essential factors collectively inhibit WNT signaling, either by reducing β-catenin levels or by inhibiting the transcriptional activation downstream of β-catenin and TCF family members. This is consistent with small-molecule inhibition of WNT/β-catenin signaling stabilizing naïve pluripotency (*30*, *31*, *37*, *38*) and indicates that WNT pathway inhibition exerts a strong effect on reprogramming even under conditions containing a glycogen synthase kinase 3 (GSK3) inhibitor. The results of our screen show that many of these factors are also required genetically during naïve PSC reprogramming and, importantly, identify the key regulators within this signaling pathway that are central to the stabilization of naïve pluripotency. Together, our genome-wide discovery screen has revealed that disrupting pathways associated with chromatin acetylation and ubiquitination, WNT signaling inhibition, and transcription factors are detrimental for naïve PSC reprogramming.

### PRC1.3 is required for naïve PSC reprogramming

PRC1.3 has not been investigated in human pluripotency or reprogramming and, in general, is understudied compared to other Polycomb complexes. Given the unexpected link between the known functions of PRC1.3 and the strong phenotype in the genetic screen, we decided to further investigate mechanisms of PRC1.3 in naïve PSC reprogramming. Furthermore, because PRC1.3 components are expressed in pluripotent cells of the human preimplantation embryo (fig. S5A), understanding PRC1.3 function is also relevant for how cells establish naïve pluripotency during early development. We used CRISPR-Cas9 to delete the core factor *PCGF3* (ranked no. 2 of 18,365 genes) in primed PSCs, which caused the dissociation of PRC1.3 (fig. S5, B to E). *PCGF3* knockout (KO) cells grew normally under primed PSC conditions with unaltered proliferation rates, and they remained undifferentiated and maintained a standard transcriptional program and expression of marker genes (fig. S5, F to K). The absence of a strong phenotype in self-renewing primed PSCs is consistent with the results of our second CRISPR-Cas9 screen, which indicated that *PCGF3* was not required for primed PSC proliferation.

**Fig. 3.**
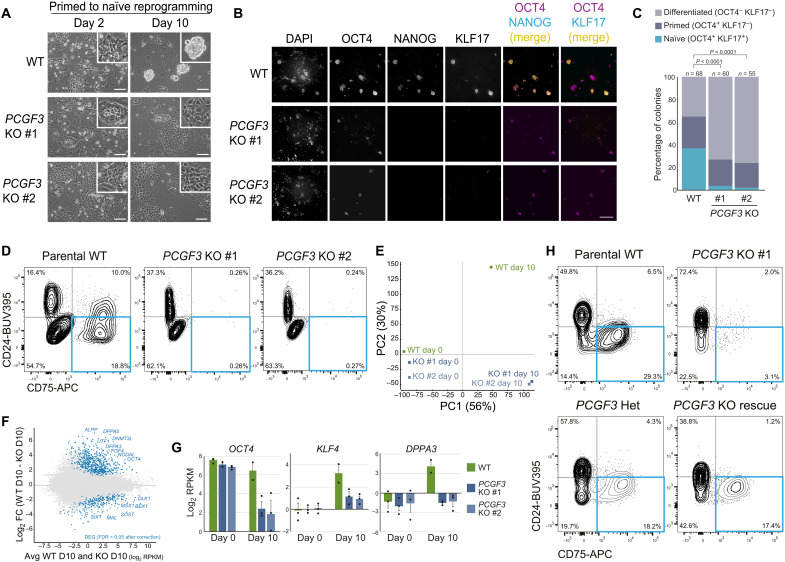
Defining PRC1.3 as an essential regulator of naïve PSC reprogramming. (**A**) Parental WT and *PCGF3* KO PSCs at days 2 and 10 of naïve PSC reprogramming (5i/L/A). Images are representative of three independent experiments. Scale bars, 100 μm. (**B**) Immunofluorescent images of WT and *PCGF3* KO cells at day 10 of naïve PSC reprogramming (5i/L/A). OCT4 and NANOG are expressed in naïve and primed PSCs; KLF17 is naïve specific. Scale bar, 400 μm. (**C**) Classification of cell colonies from (B). Number of counted colonies (*n*). The proportion of colony type (naïve, primed, or differentiated) is dependent on whether the culture is WT or *PCGF3* KO (chi-square test; 2 df; *P* < 0.0001 for WT versus each KO). (**D**) Flow cytometry of parental WT and *PCGF3* KO PSCs at day 14 of naïve PSC reprogramming (5i/L/A). Reprogrammed naïve cells in bottom right quadrant. Data are representative of three independent experiments. (**E**) RNA-seq principal components analysis (PCA) for parental WT and *PCGF3* KO PSCs at days 0 and 10 of naïve PSC reprogramming (5i/L/A). Samples from bulk cell populations. (**F**) MA plot of RNA-seq compares WT and *PCGF3* KO cell populations at day 10 of naïve PSC reprogramming (5i/L/A). Differentially expressed genes (DEG), blue (FDR < 0.05; Wald test with Benjamini-Hochberg correction). Genes higher in WT include naïve PSC and pan-pluripotency markers. Genes higher in KO include neural markers. Data show average of two (WT) or three (KO) biological replicates. (**G**) Expression of pan-pluripotency gene *OCT4* and naïve PSC markers *KLF4* and *DPPA3*. Error bars, SEM; circles show biological replicates (*n* = 2 for WT day 10; *n* = 3 for all other samples). (**H**) Flow cytometry of parental WT, *PCGF3* heterozygous (Het), *PCGF3* KO, and *PCGF3* KO expressing *PCGF3* transgene (Rescue) PSCs at day 10 of naïve PSC reprogramming (CR conditions). Data are representative of two KO lines and two independent experiments.

We next initiated primed to naïve PSC reprogramming using 5i/L/A conditions in two *PCGF3* KO cell lines and parental wild-type (WT) cells. WT cells produced characteristic naïve PSC colonies after 10 days of reprogramming, whereas, in contrast, *PCGF3* KO PSCs produced flattened, dispersed colonies that resembled neither primed nor naïve PSCs (Fig. 3A). Corroborating these morphological differences, immunofluorescence microscopy confirmed that very few colonies in the KO cultures expressed the naïve PSC marker KLF17, and a greater proportion of the colonies were differentiated (Fig. 3, B and C). Flow cytometry analysis of multiple pluripotent state cell surface markers (*32*, *39*–*41*) showed that the proportion of reprogrammed naïve PSCs in the cell population was substantially lower in the KO cells (<1%) compared to WT cells (~19% CD75^+^/CD24^−^; Fig. 3D; ~29% CD75^+^/SUSD2^+^; fig. S5L). The KO cells were not simply delayed in reprogramming because we observed the same phenotype after 24 days of reprogramming (fig. S5M), and after 60 days of continuous culture, there were no cells remaining in the KO cultures.

Consistent with this notable phenotype, RNA-seq analysis of bulk populations showed that the transcriptional profiles of WT and *PCGF3* KO cells differed substantially at day 10 of reprogramming (Fig. 3E). Differential gene expression analysis revealed that naïve pluripotency marker genes were up-regulated during reprogramming in WT cells but not in *PCGF3* KO cells (Fig. 3, F and G, and fig. S5N). Furthermore, decreased levels of pan-pluripotency markers and induction of differentiation genes indicate that the KO cells have exited the pluripotent state under these conditions (Fig. 3, F and G). To determine whether the reprogramming phenotype extended to alternative methods of naïve PSC reprogramming, we tested our cell lines under chemical resetting (CR) conditions (*30*). Under these conditions, *PCGF3* KO cells were also severely compromised in their ability to reprogram into a naïve state compared to WT cells (Fig. 3H and fig. S5, O and P). As additional controls, we initiated reprogramming with *PCGF3* heterozygous cells and with *PCGF3* KO cells that expressed a *PCGF3* transgene (fig. S5Q). These PSC lines had a much higher proportion of nascent naïve cells in the population compared to the *PCGF3* KO PSCs, indicating that reprogramming ability was restored (Fig. 3H and fig. S5, O and P). Last, deletion of an alternative PRC1.3 component, *FBRS*, also resulted in cells that generated a strongly reduced proportion of reprogrammed cells compared to WT cells analyzed at the same time point, as predicted by our screen (fig. S6, A to E). Together, these results establish a critical new role for PRC1.3 in reprogramming human cells into a naïve state.

### Misregulation of PRC1.3 target genes is associated with a failure of naïve reprogramming

**Fig. 4.**
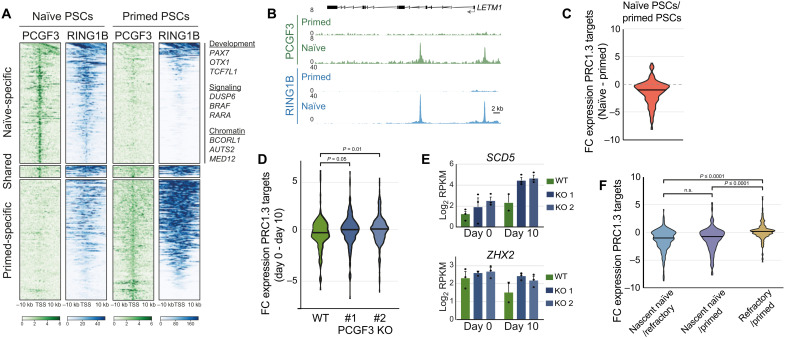
PRC1.3 represses target genes to ensure naïve PSC reprogramming. (**A**) Heatmaps of normalized PCGF3 and RING1B ChIP-seq read counts within a 20-kb transcriptional start site (TSS)–centered window in naïve and primed PSCs. Regions were subsetted into naïve-specific PRC1.3 sites (*n* = 204), shared PRC1.3 sites (*n* = 19), and primed-specific PRC1.3 sites (*n* = 205). Genes bound by PRC1.3 only in naïve PSCs are enriched for transcriptional regulators, and several example categories and genes are shown. (**B**) ChIP-seq genome browser tracks over the *LETM1* locus exemplifies a change in PRC1.3 (PCGF3 and RING1B) occupancy between naïve and primed PSCs. (**C**) Violin plot shows the fold change (FC) in the expression of PRC1.3 target genes (*n* = 191) in naïve compared to primed PSCs (average values of three biological replicates for each cell type). Central line, median. (**D**) Violin plots show the FC in the expression of PRC1.3 target genes (*n* = 191) between WT and *PCGF3* KO cells at day 0 compared to day 10 of naïve reprogramming (5i/L/A conditions; *n* = 2 for WT day 10; *n* = 3 for all other samples). *P* values were calculated using a Kruskal-Wallis test with Dunn’s multiple comparison test. Central line, median. (**E**) Charts show log_2_ RPKM (Reads Per Kilobase of transcript per Million mapped reads) for two PRC1.3 target genes in WT and *PCGF3* KO cell populations at days 0 and 10 of naïve PSC reprogramming. Error bars, SEM; circles show each biological replicate (*n* = 2 for WT day 10; *n* = 3 for all other samples). (**F**) Violin plots show the FC in the expression of PRC1.3 target genes (*n* = 191) in cell-sorted populations at day 10 of naïve reprogramming [5i/L/A conditions; data from (*32*)]. Average of three biological replicates for each sample. *P* values were calculated using a Kruskal-Wallis test with Dunn’s multiple comparison test. Central line, median. n.s., not significant.

To investigate the role of PRC1.3 in reprogramming, we used Chromatin immunoprecipitation sequencing (ChIP-seq) in naïve and primed PSCs to identify a stringent set of genes that are co-occupied by two core components, PCGF3 and RING1B (Fig. 4, A and B). Nearly all target genes (96%) were specific to either naïve or primed PSCs, indicating that PRC1.3 undergoes substantial changes in occupancy between the two pluripotent states (Fig. 4, A and B, and fig. S7, A and B). PRC1.3 target genes in naïve PSCs are enriched for transcriptional regulators, including key factors in developmental, signaling, and chromatin pathways (Fig. 4A).

The majority of genes (>80%) that retained or gained PRC1.3 occupancy during primed to naïve reprogramming were down-regulated during reprogramming (Fig. 4C and fig. S7C). RNA-seq of reprogramming cells revealed that many PRC1.3 target genes were aberrantly expressed in *PCGF3* KO cells, whereas these genes were repressed in WT cells (Fig. 4, D and E, and fig. S7D). Furthermore, cell sorting and RNA-seq revealed that PRC1.3 target genes were transcriptionally down-regulated in nascent naïve cells compared to primed PSCs but were not down-regulated in refractory cells that failed to reprogram (Fig. 4F). Together, these results show that PRC1.3 targets a cohort of developmental and signaling factors for transcriptional repression and that the inability to silence these genes is associated with the failure to reprogram cells into a naïve state.

### Pluripotent state–specific PRC1.3 composition

**Fig. 5.**
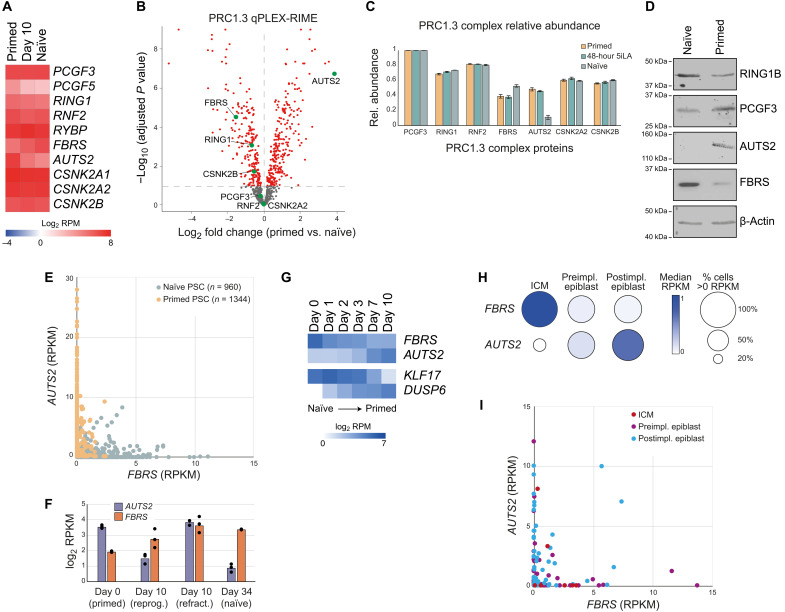
FBRS and AUTS2 paralogs switch during human development. (**A**) Expression of PRC1.3 components at day 0 (primed PSCs), day 10 (nascent naïve PSCs), and day 34 (established naïve PSC lines). Data from (*32*). (**B**) FC in abundance of PCGF3-interacting proteins when comparing naïve and primed PSCs. PRC1.3 components are labeled. Adjusted *P* < 0.1, red dots; adjusted *P* > 0.1, gray dots (Limma test with Benjamini-Hochberg correction). Biological replicates: *n* = 4 primed; *n* = 2 naïve. (**C**) Relative abundance of PRC1.3 components in naïve, 48-hour 5i/L/A, and primed PSCs, measured using qPLEX-RIME analysis of PCGF3-interacting proteins. Mean with SD; biological replicates: *n* = 4 primed; *n* = 4 48-hour 5i/L/A; *n* = 2 naïve. (**D**) Expression of PRC1.3 proteins in naïve and primed PSCs. Naïve PSCs (WA09) in t2i/L+PKCi and primed PSCs (WA09) in KSR-containing medium. (**E**) Expression levels of *FBRS* and *AUTS2* in single cells; <2% cells have RPKM > 1 for both genes. Naïve PSCs, blue; primed PSCs, orange. Data from (*66*). (**F**) Expression of *AUTS2* and *FBRS* in primed PSCs, nascent naïve PSCs (day 10, reprog.), refractory cells (day 10, refract.), and established naïve PSC lines. Individual replicates shown (*n* = 3). Data from (*32*). (**G**) Expression of *FBRS* and *AUTS2* over a naïve (day 0) to primed (day 10) capacitation time course. Naïve marker *KLF17* and primed marker *DUSP6* are also shown. Data from (*67*). (**H**) *FBRS* and *AUTS2* expression in pluripotent cells over three stages of human development. Circle color, median expression level of single cells; circle size, percentage of single cells with an RPKM value > 0. ICM, inner cell mass (*n* = 10); preimpl. epiblast, preimplantation epiblast cells (*n* = 61); postimpl. epiblast, postimplantation epiblast cells (*n* = 64). Data and cell annotations from (*68*). (**I**) Expression of *FBRS* and *AUTS2* in single cells during human development; <8% cells have RPKM > 1 for both genes. Data and cell annotations from (*68*).

The expression levels of most PRC1.3 complex components do not change during naïve reprogramming (Fig. 5A). We therefore sought to determine whether the composition of PRC1.3 differs between naïve and primed PSCs. We used quantitative, multiplexed rapid immunoprecipitation mass spectrometry (MS) of endogenous protein (qPLEX-RIME) (*42*, *43*) to identify proteins that interact on chromatin with PCGF3, which is a component that is specific to PRC1.3 (fig. S7E and data S3) (*44*, *45*). Known PRC1.3 complex proteins were identified in primed PSCs, and those components were much less abundant in primed PSCs that lack PCGF3 and PCGF5, thereby confirming the specificity of the assay (fig. S7F). Comparing PCGF3-associated proteins between naïve and primed PSCs showed that the relative abundance of most PRC1.3 components was similar between the two cell types (Fig. 5, B and C). Unexpectedly, however, the abundance of two PRC1.3 paralog proteins, Fibrosin (FBRS) and Activator of transcription and developmental regulator (AUTS2), differed, whereby AUTS2 interacted with PCGF3 in primed but less in naïve PSCs and vice versa for FBRS (Fig. 5, B and C). We initially hypothesized that the paralog switch occurs in response to the signaling inhibitors within the reprogramming cocktail. However, analysis of PCGF3-interacting proteins in primed PSCs that were cultured in 5i/L/A for 48 hours showed that AUTS2 was still the more abundant PRC1.3 paralog (Fig. 5C). Instead, this switch is likely to be driven by paralog availability, as FBRS is more abundant in naïve PSCs and then transitions to AUTS2 as the dominant paralog in primed PSCs (Fig. 5, A and D). Individual naïve and primed cells express either *FBRS* or *AUTS2*, respectively, with few cells (<2%) coexpressing both paralogs (Fig. 5E). By day 10 of reprogramming, *AUTS2* transcript levels were strongly reduced in the nascent naïve cells but remained highly expressed in refractory cells that failed to reprogram (Fig. 5F). Conversely, *FBRS* is moderately up-regulated both in nascent naïve PSCs and in refractory cells (Fig. 5F). This switch in paralog expression is consistent with the results of our prior screen, which identified an essential role in naïve reprogramming for *FBRS* (ranked no. 10), but *AUTS2* was not required (ranked no. 14,751) (Fig. 2E).

Transcriptional analysis of naïve to primed PSC transition revealed a changeover from *FBRS* to *AUTS2* expression (Fig. 5G). *FBRS* and *AUTS2* also showed anticorrelated expression patterns during human embryo development; *FBRS* is more highly expressed in early epiblast and then switches over to *AUTS2* as the more abundant paralog in postimplantation epiblast cells (Fig. 5H). Similar expression patterns are also observed in cynomolgus monkey development (fig. S7G). Moreover, most of the individual epiblast cells expressed either *FBRS* or *AUTS2* but rarely both genes together (Fig. 5I). Thus, the *FBRS* to *AUTS2* paralog switch occurs in epiblast cells during embryo implantation and is recapitulated in naïve to primed PSCs transitions. Curiously, several *AUTS2* transcriptional start sites are bound by PRC1.3 in naïve but not in primed PSCs (fig. S7H), which is consistent with the low levels of *AUTS2* in naïve PSCs (Fig. 5, D and G). This suggests that there could be an interesting self-regulation of the two paralogs involving PRC1.3 itself. Together, these results establish that, although the composition of PRC1.3 is largely retained between naïve and primed PSCs, there is a paralog switch between *FBRS* and *AUTS2* that occurs upon pluripotent state transitions and also during the implantation phase of human development.

### PRDM14 interacts with PRC1.3 to ensure naïve cell reprogramming and gene regulation

**Fig. 6.**
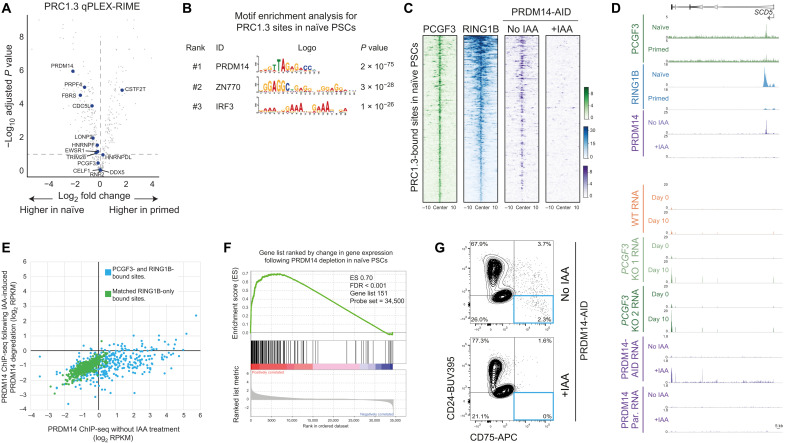
PRC1.3 and PRDM14 associate on chromatin to repress target genes and ensure naïve PSC reprogramming. (**A**) qPLEX-RIME data show FC in abundance of PCGF3-interacting proteins. All proteins in dataset, gray dots; factors essential for naïve reprogramming, blue circles. (**B**) Motif enrichment for PRC1.3 target sites in naïve PSCs. Top three motifs shown, ranked by adjusted *P* value (Fisher’s exact test with Bonferroni correction). (**C**) Normalized ChIP-seq read counts within a 20-kb peak-centered window in naïve PSCs. Peaks correspond to PRC1.3-bound sites in naïve PSCs (*n* = 378). PRDM14 heatmaps show ChIP-seq signal under normal [no indole-3-acetic acid (IAA)] and PRDM14-depleted (+IAA) conditions. (**D**) ChIP-seq (top 6 tracks) and RNA-seq (lower 10 tracks) at an example PRC1.3 and PRDM14 target gene. *SCD5* expression is low in WT and *PCGF3* KO primed PSCs (day 0) and remains low in WT cells after 10 days of 5i/L/A reprogramming. In contrast, *SCD5* is derepressed in *PCGF3* KO PSCs at day 10 of reprogramming, as well as in 4i-cultured PSCs following IAA-induced degradation of PRDM14-AID. Treating parental (Par.) cells that lack the AID tag with IAA has no effect. PRDM14 ChIP-seq and RNA-seq data from (*47*). (**E**) ChIP-seq normalized read counts without IAA and following IAA-induced PRDM14 degradation. Blue circles, PRC1.3 target sites (*n* = 378); green circles, RING1B-only sites with matched distribution of RING1B ChIP-seq levels (*n* = 397). Data from (*47*). (**F**) Gene set enrichment analysis (GSEA) of naïve PRC1.3 target genes ranked by FC in transcription following IAA-induced degradation of PRDM14 in naïve PSCs. Genes derepressed in the absence of PRDM14 are enriched in PRC1.3 targets (FDR < 0.001; Kolmogorov-Smirnov test). (**G**) Flow cytometry of PRDM14-AID-VENUS PSCs at day 10 of naïve PSC reprogramming (CR conditions) in the absence (top) or presence (bottom) of IAA-induced PRDM14 degradation. Successfully reprogrammed naïve cells in the bottom right quadrant. Data are representative of three independent experiments.

We next investigated the regulation of PRC1.3 in human pluripotent states. The qPLEX-RIME data revealed differences in PRC1.3 interactions on chromatin between the two cell types (Fig. 6A and data S3). This included naïve-enriched proteins, such as the linker DNA binding histone protein HISTH1.1, the DNA methyltransferase regulator DNMT3L, and the transcription factor PR/SET domain 14 (PRDM14) (Fig. 6A). PRDM14 is a central regulator of pluripotency (*46*, *47*) and was of particular interest because, similar to PRC1.3, it was also essential for naïve PSC reprogramming (ranked no. 74) (Fig. 6A and fig. S4D).

We confirmed the interaction between PRC1.3 and PRDM14 in naïve PSCs by coimmunoprecipitation (Co-IP) (fig. S8A). This association was further supported by motif analysis of PRC1.3 peaks in naïve PSCs, which revealed that the PRDM14 motif was the highest enriched motif at PRC1.3 peaks in naïve PSCs (Fig. 6B), whereas the PRDM14 motif was not enriched at PRC1.3 peaks in primed PSCs or in regions matched for GC content. Moreover, in naïve PSCs, the proportion of PRC1.3 peaks containing a PRDM14 motif was significantly higher compared to RING1B sites that lacked PCGF3 occupancy (53% versus 6%; *P* < 0.0001, two-sided Fisher’s exact test) and to regions matched for GC content (53% versus 5%; *P* < 0.001, two-sided Fisher’s exact test). Analysis of ChIP-seq data (*47*) showed that PRDM14 was bound at the majority (72%) of PRC1.3 peaks in PSCs cultured in 4i naïve medium, and the ChIP signal was reduced to background levels following the induced degradation of PRDM14-AID-VENUS (Fig. 6, C and D) (*47*). PRDM14 occupancy was significantly higher at PRC1.3 peaks compared to RING1B-only bound sites (Fig. 6E and fig. S8B), which further supports a specific association between PRDM14 and PRC1.3. Furthermore, PRC1.3 target genes were transcriptionally derepressed following the acute depletion of PRDM14 in naïve PSCs (Fig. 6, D and F, and fig. S8, C and D). Last, we initiated naïve cell reprogramming of primed PRDM14-AID-VENUS PSCs (fig. S8, E and F) (*47*). The ability to reprogram into the naïve state was low for this cell line even in the presence of PRDM14-AID-VENUS (Fig. 6G), potentially because of impaired function of the fusion protein. Nevertheless, no nascent naïve PSCs were obtained when PRDM14 was degraded during reprogramming, which demonstrates that PRDM14 is required for naïve cell reprogramming (Fig. 6G). Together, these results establish that PRDM14 and PRC1.3 function together to control target gene repression and to ensure the reprogramming of cells into naïve pluripotency.

### Overcoming reprogramming impediments to improve naïve PSC reprogramming

**Fig. 7.**
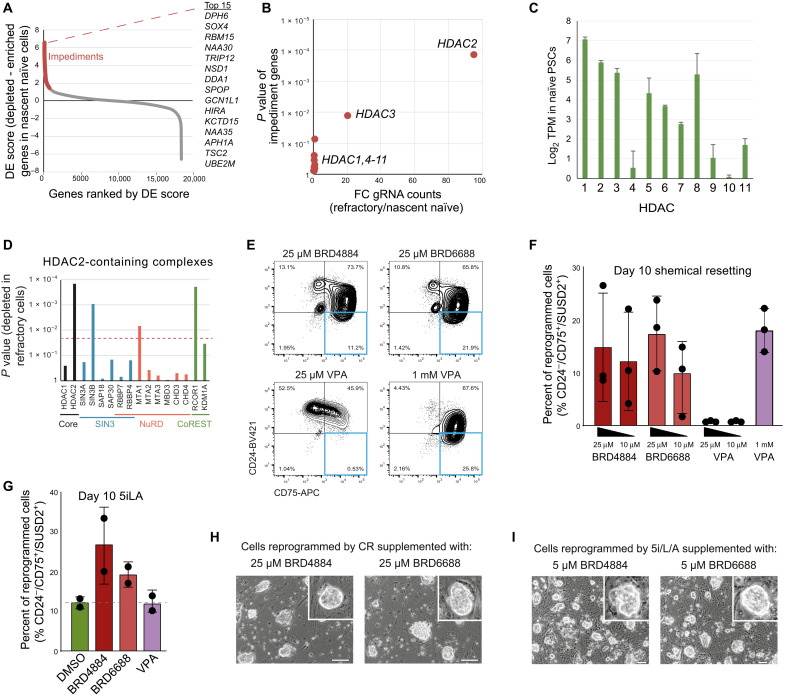
Inhibiting HDAC2 improves naïve PSC reprogramming efficiency using multiple methods. (**A**) Ranked DE plot by comparing nascent naïve with refractory populations. Impediment genes are highlighted. The top 15 ranked impediment genes are shown. (**B**) Chart shows the FC in gRNA counts and associated *P* values for all HDAC genes. (**C**) Chart shows the expression levels of each HDAC gene in naïve PSCs. Mean with SD, *n* = 3 biological replicates. Data are from (*32*). TPM, Transcripts Per Million. (**D**) Chart shows the *P* values of HDAC2-containing complex members as a measure of their depletion in the refractory cell population following the CRISPR-Cas9 screen. The red line indicates *P* = 0.02 as a significance cutoff. (**E**) Flow cytometry contour plots of WT PSCs at day 10 of naïve PSC reprogramming (CR conditions). HDAC2 inhibitors BRD4884 and BRD6688 and pan-HDAC inhibitor VPA were added at 25 μM. VPA added at 1 mM is also shown, which is the concentration used in the CR protocol and serves as a positive control. Successfully reprogrammed naïve cells appear in the bottom right quadrant. Data are representative of three independent experiments. (**F**) Chart shows the summary results from (E). Data show mean with SD for three independent experiments. Note that there was no significant difference in the reprogramming efficiency between the HDAC2 inhibitor–treated cells and the cells treated with VPA at 1 mM (two-tailed *t* test). (**G**) Chart shows the percentage of nascent naïve cells as measured by flow cytometry after 10 days of reprogramming under 5i/L/A conditions. HDAC2 inhibitors BRD4884 and BRD6688 and pan-HDAC inhibitor VPA were supplemented to the media at 5 μM for the first 3 days. Data show mean with SD for two independent experiments. DMSO, dimethyl sulfoxide. (**H** and **I**) Phase-contrast images of naïve PSCs following reprogramming in (H) CR medium or (I) 5i/L/A medium, supplemented with HDAC2 inhibitors BRD4884 or BRD6688. Scale bars, 100 μm.

We next investigated the genes that impede naïve PSC reprograming that were identified in our CRISPR-Cas9 screen (Fig. 7A). Our genetic screen identified *HDAC2* as a strong impediment of naïve PSC reprogramming (ranked no. 40 of 18,365 impediment genes); cells targeted with *HDAC2* gRNAs were 100-fold enriched in the successfully reprogrammed cell population compared to refractory cells (Fig. 7B). The deletion of the other histone deacetylase (HDAC)–encoding genes had little impact on naïve PSC reprogramming (Fig. 7B) despite being expressed at similar levels (Fig. 7C). HDAC2 is a component of three distinct complexes, Swi-independent 3 (SIN3), Nucleosome remodelling and deacetylase (NuRD), and Cofactors of repressor element-1 silencing transcription factor (CoREST). Results from our CRISPR screen show that most members of the SIN3 and NuRD complexes can be deleted without affecting naïve PSC reprogramming (Fig. 7D). In contrast, members of the CoREST complex, RCOR1 (ranked no. 42 in the list of impediments; *P* = 0.0002, permutation test) and KDM1A (ranked no. 815 in the list of impediments; *P* = 0.03, permutation test), are promising candidates that impede naïve PSC reprogramming and whose targeted deletion led to enhanced reprogramming (Fig. 7D).

The discovery of HDAC2 as a major reprogramming impediment is important because the treatment of cells with pan-HDAC inhibitors increases reprogramming and transdifferentiation efficiency in many contexts, including toward naïve PSCs, although it is unknown which HDACs elicit this effect (*30*, *48*–*50*). We therefore tested whether the targeted inhibition of HDAC2 could facilitate naïve PSC reprogramming. We first examined whether the broad-spectrum HDAC inhibitor valproic acid (VPA), a component of CR medium, could be replaced with selective HDAC2 inhibitors BRD4884 and BRD6688 (*51*). Both HDAC2 inhibitors generated nascent naïve cells with high efficiencies, and the HDAC2 inhibitors were effective at 100-fold lower concentrations than VPA (Fig. 7, E and F). We additionally found that HDAC2 inhibitors were also effective under alternative reprogramming conditions. We supplemented 5i/L/A medium with BRD4884, BRD6688, or VPA at equal concentrations for the first 3 days of reprogramming. At day 10 of reprogramming, the proportion of nascent naïve PSCs in the cell population following HDAC2 inhibition was two- to threefold higher than VPA (Fig. 7G). The naïve cells generated by HDAC2 inhibitor treatment were propagated and formed stable cell lines (Fig. 7, H and I). These results establish that supplementation with HDAC2 inhibitors is of strong practical benefit that can improve on current reprogramming methods.

## DISCUSSION

Here, we identify a comprehensive set of regulators that control the establishment of naïve pluripotency in human cells. Our study has uncovered crucial complexes and pathways that are essential for primed to naïve cell reprogramming and, additionally, those that create barriers to impede this process. The results describe the first genome-wide CRISPR-Cas9–based functional screen in human cell reprogramming and provide an important new dataset that can be mined by the scientific community to understand the processes controlling human pluripotent cell identity.

Our screen led us to identify an essential new role for the noncanonical Polycomb repressive complex PRC1.3 in naïve PSC reprogramming. Expanding upon this finding, we dissected the mechanism whereby PRC1.3 and PRDM14 transcriptionally repress a set of developmental, chromatin, and signaling regulators and that the failure to silence these factors is associated with a failure to reprogram. It is likely that not all PRC1.3 target genes have detrimental effects, but having identified this gene set, these candidates can be examined in further studies as potential disruptors of cell reprogramming. PRC1.3 was required for naïve reprogramming under different conditions (5i/L/A and CR). This indicates that each condition could transition cells using similar regulatory pathways, which is consistent with their similar transcriptional trajectories during reprogramming (*29*). Several of the PRC1.3 target genes are associated with neural differentiation, and these genes had elevated expression in refractory cells and also when PRC1.3 was inactivated. A previous study showed that most cells that fail to reprogram to naïve pluripotency adopt a neural phenotype (*32*). PRC1.3, therefore, might serve as a transcriptional repressor to silence alternative lineage fates, and potentially changing the strength of this repression can alter the balance either toward successful naïve cell reprogramming or to refractory cells. This hypothesis is supported by the high ranking of several neural determinants as reprogramming impediments. Together, these results suggest that blocking alternative routes might help channel cells along the correct path. This has important implications for the improved design of reprogramming strategies.

Our study also uncovered new insights into PRC1.3 regulation and function. Unusually for a PRC1 complex, PRC1.3 lacks an auxiliary component that binds directly to DNA sequences or to specific chromatin modifications. Instead, our results lead us to propose that PRDM14, a pluripotency transcription factor, recruits PRC1.3 to specific target sites during cell reprogramming. This proposal is supported by the specific association between PRC1-occupied sites and PRDM14 binding and motif enrichment, together with the interaction between PRC1.3 and PRDM14 in the absence of DNA and also when bound to chromatin. It is currently unclear whether the association between PRC1.3 and PRDM14 is facilitated by the higher levels of PRDM14 in naïve compared to primed PSCs or alternatively by changes to the composition or modifications to PRC1.3 that could potentially enable this interaction. Our findings extend prior work showing that PRDM14 recruits an alternative Polycomb repressive complex, PRC2, to stabilize naïve pluripotency in mouse (*52*, *53*). In mouse embryonic stem cells, the DNA binding protein Upstream transcription factor 1 (USF1) could have a similar role as an auxiliary factor for PRC1.3 recruitment (*54*). In contrast to our study, however, USF1-PRC1.3 was associated with gene activation rather than repression (*54*); therefore, different recruitment mechanisms and subunit composition may endow PRC1.3 with different functional properties (*55*). PRC1.3 could also have additional roles that are independent of developmental gene regulatory control including low-level genome-wide coverage to constrain transcriptional activation or associations with noncoding RNAs (*54*, *56*, *57*). Examining these possibilities in the context of pluripotent state transitions is an exciting area for future investigation.

We also uncovered a developmentally controlled switch that occurs between the paralogs *FBRS* and *AUTS2*, whereby FBRS-containing PRC1.3 is the dominant complex in naïve PSCs and in preimplantation human embryos. Consistent with this expression profile, *FBRS*, but not *AUTS2*, is required for naïve cell reprogramming. Previous studies have shown that the integration of AUTS2 within a related complex, PRC1.5, is associated with transcriptional activation (*55*, *58*); however, PRC1.3-AUTS2 in primed PSCs does not seem to be associated with activation. This difference could potentially be due to the expression of different *AUTS2* isoforms, which have distinct and incompletely understood roles in transcriptional repression and activation (*59*). Protein interaction studies have identified FBRS and AUTS2 as components of PRC1.3 (*44*, *45*), but it was not known that their association with PRC1.3 could differ or that each version of the complex could have distinct and separable requirements. This finding underscores the remarkable and dynamic variation in the composition of PRC1 complexes to adapt to specific roles. Further examination of PRC1.3 during the transition from naïve to primed PSCs could serve as a paradigm for how cell type–specific variants of chromatin-modifying complexes act to control cell state changes. In particular, an important future challenge remains in understanding whether the switch between FBRS and AUTS2 paralogs endows PRC1.3 with distinct biochemical or functional roles.

Naïve PSCs harbor desirable and unique properties, including a seemingly unrestricted developmental potential encompassing the ability to efficiently generate hypoblast and trophoblast cell types including human blastoids (*17*–*24*), in addition to serving as a model for peri-implantation developmental processes, such as the reconfiguration of the epigenome (*11*, *12*). Progress in this area has been hampered because of an inadequate understanding of the mechanisms that control naïve pluripotency and also restricted by suboptimal growth conditions. We anticipate that the new insights obtained in this study should lead to improved reprogramming methods that are designed to overcome specific impediments with minimal impact on nontarget pathways. As a first step, we demonstrated the feasibility of this approach by identifying HDAC2 as a key reprogramming impediment and show that replacing a broad-spectrum HDAC inhibitor with a selective HDAC2 inhibitor is of strong practical benefit that can improve on current reprogramming conditions. Advances based on this and other reprogramming impediments identified here should facilitate the increased use and exploitation of naïve PSCs in research and clinical applications.

There are several limitations that are associated with the genome-wide CRISPR-Cas9 screen. First, our screen was designed to compare the sgRNA counts between the nascent naïve cell population and the refractory population, but a blind spot of this is that gene KOs that are depleted before reprogramming would be missed. It is possible that some of those genes could have roles in cell reprogramming that we would not detect. Second, the screen is unable to identify genes that have cell nonautonomous effects, as any consequence of deleting this type of gene would be masked by most of the cells in the population that retain the gene’s function. Third, this type of genetic screen can be affected by clonal or proliferation effects that could lead to changes in sgRNA counts when comparing between cell populations. This effect is a limitation of the study and one that we tried to mitigate by minimizing the reprogramming duration.

Together, we have uncovered a comprehensive set of factors involved in naïve human PSC reprogramming. These findings provide new insights into the principles controlling human pluripotent states and have established a new role for variant Polycomb complexes in pluripotent state transitions. We anticipate that these discoveries will lead to the increased use and exploitation of human naïve PSCs including the advancement of human embryo models.

## MATERIALS AND METHODS

Reagent and resource details are provided in data S4.

### Cell culture

WA09/H9 primed PSCs were obtained from WiCell. WA09/H9 NK2 naïve PSCs were provided by A. Smith (*25*) with permission from WiCell. WA09/H9 PRDM14-AID-VENUS primed cells were provided by A. Surani (*47*), and FSPS13B primed iPSCs were provided by L. Vallier (*60*). H9 is a female (XX) cell line, and FSPS13B is a male (XY) cell line. For most of the experiments, PSCs were cultured in 5% O_2_ and 5% CO_2_ at 37°C. To generate the FSPS13B CAGCAS9 B1 primed PSC line and for the primed PSC proliferation screen, cells were cultured in 20% O_2_ and 5% CO_2_ at 37°C.

WA09/H9 primed PSCs were cultured under either feeder-dependent or feeder-free conditions. Feeder-dependent conditions (*3*) were grown in 80% advanced Dulbecco’s modified Eagle’s medium (DMEM), 20% KnockOut Serum Replacement (KSR), 2 mM l-glutamine, penicillin-streptomycin (50 U/ml), 0.1 mM β-mercaptoethanol (all from Thermo Fisher Scientific), and basic fibroblast growth factor (4 ng/ml; Wellcome–MRC Cambridge Stem Cell Institute) on irradiated mouse embryonic fibroblasts (MEFs) seeded at a density of 1 million cells per six-well plate. Cells were passaged by 5-min incubation with collagenase type IV (200 U/ml) at 37°C (Thermo Fisher Scientific). For feeder-independent conditions, cells were transferred onto Vitronectin-coated plates (0.5 μg/cm^2^; Thermo Fisher Scientific) in complete TeSR-E8 or mTeSR1 medium (STEMCELL Technologies). Cells were passaged by 5-min incubation at room temperature with 0.5 mM EDTA in phosphate-buffered saline (PBS).

Naïve PSCs were cultured under titrated 2i (CHIR99021 and PD0325901 inhibitors) with Leukaemia Inhibitory Factor and Protein Kinase C inhibitor conditions (*25*) in a 1:1 mixture of DMEM/F12 and Neurobasal, 0.5× N-2 supplement, 0.5× B-27 supplement, 2 mM l-glutamine, penicillin-streptomycin (50 U/ml), 0.1 mM β-mercaptoethanol (all from Thermo Fisher Scientific), 1 μM PD0325901, 1 μM CHIR99021, human leukemia inhibitory factor (LIF) (20 ng/ml) (all from Wellcome–MRC Cambridge Stem Cell Institute), and 2 μM Gö6983 (PKCi; Tocris) on a MEF layer seeded at a density of 2 million cells per six-well plate. For feeder-free culture, t2i/L+PKCi naïve PSCs were cultured on Matrigel-coated plates (Corning). Cells were passaged by 5-min incubation at 37°C with Accutase (BioLegend). For PRDM14-AID-VENUS depletion in naïve PSCs, indole-3-acetic acid (IAA) was applied at 100 μM for 24 hours.

For primed to naïve PSC reprogramming under 5i/L/A conditions (*26*), primed PSCs were dissociated into single cells with Accutase, and 1.2 million cells per 10-cm tissue culture dish were plated in primed PSC medium with 10 μM Y-27632 (Cell Guidance Systems) onto MEF seeded at a density of 4 million cells per 10-cm dish. The following day, medium was changed to 5i/L/A medium composed of a 1:1 mixture of DMEM/F12 and Neurobasal, 0.5× N-2 supplement, 0.5× B-27 supplement, 2 mM l-glutamine, penicillin-streptomycin (50 U/ml and 50 μg/ml), 0.1 mM β-mercaptoethanol (all from Thermo Fisher Scientific), bovine serum albumin (BSA; 50 μg/ml; Thermo Fisher Scientific), 0.5% KSR (Thermo Fisher Scientific), recombinant human LIF (20 ng/ml), activin A (20 ng/ml), 1 μM PD0325901 (all from Wellcome–MRC Cambridge Stem Cell Institute), 1 μM IM-12, 1 μM WH-4-023, 0.5 μM SB590885, and 10 μM Y-27632 (all from Cell Guidance Systems). Cells were passaged with Accutase on days 5 and 10 and then every 4 days. For HDAC inhibitor experiments, 5i/L/A medium was supplemented with either BRD4884, BRD6688, or VPA at 5 μM for the first 3 days of reprogramming.

For primed to naïve PSC reprogramming under CR conditions (*30*), primed PSCs were dissociated into single cells with Accutase, and 1.2 million cells per 10-cm tissue culture dish were plated in primed PSC medium containing 10 μM Y-27632 at a density of 4 million cells per 10-cm dish. The following day, medium was changed to Chemical Reset Media 1 (cRM-1) composed of a 1:1 mixture of DMEM/F12 and Neurobasal, 0.5× N-2 supplement, 0.5× B-27 supplement, 2 mM l-glutamine, penicillin-streptomycin (50 U/ml), 0.1 mM β-mercaptoethanol (all from Thermo Fisher Scientific), 1 μM PD0325901, human LIF (10 ng/ml; Wellcome–MRC Cambridge Stem Cell Institute), and 1 mM VPA (Sigma-Aldrich). Medium was replaced daily. On day 5, cells were passaged with Accutase, and the medium was changed to cRM-2 composed of 1:1 mixture of DMEM/F12 and Neurobasal, 0.5× N-2 supplement, 0.5× B-27 supplement, 2 mM l-glutamine, penicillin-streptomycin (50 U/ml), 0.1 mM β-mercaptoethanol (all from Thermo Fisher Scientific), 1 μM PD0325901, human LIF (10 ng/ml; Wellcome–MRC Cambridge Stem Cell Institute), 2 μM Gö6983 (Tocris), and 2 μM XAV939 (Sigma-Aldrich). Medium was changed daily thereafter. Cells were subsequently passaged with Accutase on day 10 and then every 4 days. For PRDM14-AID-VENUS depletion during CR, IAA sodium salt was applied at 100 μM from 48 hours of reprogramming onward. For HDAC inhibitor experiments, 1 mM VPA in cRM-1 medium was replaced with either 10 or 25 μM BRD4884, BRD6688, or VPA for the first 3 days of reprogramming.

#### Genome-wide CRISPR KO screens

Cas9, driven by the CAG promoter, was integrated into the *AAVS1* locus in the FSPS13B primed iPSC line to produce a FSPS13B CAGCAS9 BP primed PSC line. To achieve this, 1 million primed iPSCs were nucleofected with 5 μg of pZFN-AAVS1-ELD (Addgene, #159297), 5 μg of pZFN-AAVS1-KRR (Addgene, #159298), and 2 μg of pAAVS1-CAG-hCAS9-neo (Addgene, #166026) plasmids. After 48 hours posttransfection, positive clones were selected with G418 (250 μg/ml). Clonal lines were expanded, and Cas9 integration was verified using junctional polymerase chain reaction (PCR) (AAVS1-GF4, CTTAGCCACTCTGTGCTGACCACTC and CAGSB-5′P2, CGTAAGTTATGTAACGCGGAACTCC for the left homology arm; bGHpA-U2, ATGCTGGGGATGCGGTGGGCTCT and AAVS1-GR3, CACAGGTGGCGCTTCCAGTGCTCAGACTAG). Cas9-expressing clonal lines were checked for genome editing activity using our Cas9 reporter system, and we selected the most stable and active cell clone. We also checked the activity before genetic screening to ensure that cells had not undergone silencing during expansion. The resultant cell line, FSPS13B CAGCAS9 B1 primed PSCs, was cultured on Vitronectin-coated plates in complete TeSR-E8 medium. To perform a screen for genes essential for primed PSC proliferation, we transduced 30 million FSPS13B CAGCAS9 B1 primed PSCs by spinfection with the human v1 CRISPR gRNA library (*61*). Seventy-two hours after transduction, cells were harvested, and a transduction efficiency of ~30% was confirmed. The cells were then selected with puromycin for 3 days and further cultured under feeder-independent conditions (TeSR-E8 on Vitronectin) by replating 50 million cells at each passage. Twenty-one days after transduction, the cells were harvested for genomic DNA extraction. To perform a screen to identify genes involved in primed to naïve reprogramming, we transduced 30 million FSPS13B CAGCAS9 B1 primed PSCs by spinfection with the human v3 CRISPR gRNA library (*62*) at an MOI of 0.3 (*63*). Seventy-two hours after transduction, cells were harvested with Accutase and pooled, and a transduction efficiency of ~30% was confirmed by flow cytometry for BFP expression. The cells were seeded onto 10-cm culture dishes at a density of 65,000 cells/cm^2^ and cultured in TeSR-E8 medium supplemented with puromycin. On day 6 after transduction, puromycin-selected cells were harvested with Accutase and confirmed by flow cytometry to be ~95% BFP positive. The short, 6-day, time window between cell transduction and the start of cell reprogramming was designed to minimize clonal or proliferation effects in the starting cell population. Fifty-one million cells were plated on to 17 culture dishes (15 cm) that were precoated with MEFs, in TeSR-E8 medium supplemented with 20% KSR and 10 μM Y-27632. The following day, cells were rinsed briefly with DMEM/F12 and changed to 5i/L/A medium. Medium was replaced daily. Cells were passaged with Accutase on day 5 of reprogramming and split at a ratio of 1 to 2.5 so that 42 culture dishes (15 cm) were seeded. Medium was replaced daily. On day 10 of reprogramming, cells were harvested with Accutase and pooled. MEFs were depleted by labeling the samples with a biotin-conjugated anti-mouse Cd90.2 antibody (clone 30-H12; BioLegend) and using Streptavidin Microbeads (Miltenyi Biotec) to specifically isolate unlabeled human cells from a MACS LS column (Miltenyi Biotec) on a QuadroMACS magnetic separator (Miltenyi Biotec). The isolated human cells were then incubated in batches with a panel of antibodies against cell surface markers, as described in the flow cytometry methods section, and cell-sorted into two populations, nascent naïve cells and refractory cells, each containing ~7 million cells. Cells were pelleted by gentle centrifugation, and samples were frozen promptly for genomic DNA isolation.

#### gRNA sequencing

Genomic DNA was isolated from the day 21 cell populations for the primed PSC proliferation screen or from the two cell-sorted fractions for the reprogramming screen using the QIAamp DNA Blood Maxi Kit (QIAGEN) or DNeasy Blood and Tissue Kit (QIAGEN), depending on input cell numbers, following the manufacturer’s instructions. The primed PSC proliferation screen was performed in triplicate. Given the substantial technical challenge of the reprogramming discovery screen, the experiment was performed once, with subsequent hits verified by individual gene KOs. gRNA amplification from genomic DNA and Illumina sequencing were performed as described previously (*63*). For the reprogramming screen, all available genomic DNA was used in the first-round PCR at 1 μg per reaction in 25 reactions to maximize the coverage.

#### Individual gene targeting

Dual gRNAs were designed using WGE (www.sanger.ac.uk/science/tools/wge) to excise an early exon that would cause a frameshift upon deletion. Sequences used for *PCGF3* were TTAGGAGAGCGTCTAGAGCCAGG and GGCACTCACCCCACGTACTGTGG; for *PCGF5*, GTTCTTCTTCAAAAACTGTTAGG and GACAATCCTATGCTTAGAAATGG; and for *FBRS*, ATAGGCATCCAGGCCCCATCTGG and GTCACTAAGCAAGTGGAACCAGG. Each gRNA sequence was incorporated into a U6 target gRNA expression vector and synthesized as a gBlock (Integrated DNA Technologies). The gRNA gBlocks were subcloned into pCR2.1-TOPO (Thermo Fisher Scientific) and verified by sequencing. Primed PSCs were dissociated into single cells using Accutase, and 2 million cells were nucleofected with 4 μg of pCas9_GFP (Addgene, #44719) and 3 μg of each gRNA expression vector. After 48 hours, 10,000 green fluorescent protein (GFP)–positive single cells were isolated by flow cytometry and seeded onto MEF in a 10-cm tissue culture dish in primed PSC medium supplemented with 10 μM Y-27632 for the first 24 hours. Individual clones were picked and expanded in 96-well plates and genotyped by PCR. Mutations were validated by DNA sequencing of TOPO cloned PCR products.

#### PCGF3 rescue cell line

RNA was extracted using an RNeasy Mini Kit (QIAGEN) and reverse-transcribed using SuperScript II Reverse Transcriptase (Thermo Fisher Scientific). The full-length *PCGF3* coding sequence was amplified from the cDNA using HotStarTaq DNA polymerase (QIAGEN) using the primer sequences ACGACGCGTGCCACCATGTTGACCAGGAAGATCAAGCTG and ATAAGAATGCGGCCGCTCACAGCAAGTCCATCTTGGGT. A 729–base pair (bp) PCR product was excised and cloned into a pCAG expression plasmid using Mlu I and Not I enzymes (Thermo Fisher Scientific). The resultant plasmid pCAG-PCGF3-ires-puro was confirmed by DNA sequencing. The plasmid was transfected into *PCGF3*-deficient primed PSCs using GeneJuice (Merck-Millipore). Cells were treated with puromycin (1 μg/ml) for 48 hours, and resistant colonies were expanded. The expression of PCGF3 in the rescue cells was confirmed by Western blot. The line was selected on the basis of having the closest expression levels to WT cells, although PCGF3 levels in the rescue cells are higher than in the WT cells.

### Characterization of cell lines

The use of human embryonic stem cells (ESCs) was carried out in accordance with approvals from the UK Stem Cell Bank Steering Committee. In this study, we used two independently derived *PCGF3* KO clonal lines, one clonal *PCGF3* heterozygous cell line, one *PCGF3* rescue cell line, one *FBRS* KO clonal line, one PRDM14-AID-VENUS cell line [all WA09/H9 Embryonic Stem Cells (ESCs)], and the FSPS13B CAGCAS9 B1 cell line (derived from FSPS13B iPSCs). Parental WT cells described in the study correspond to untargeted parental WA09/H9 cells.

All cell lines used in this study were authenticated and confirmed to be mycoplasma negative. Pluripotent cell state and undifferentiated status were validated by protein marker expression. *PCGF3* KO, *FBRS* KO, and PRDM14-AID-VENUS primed cells expressed OCT4 and NANOG and were >~95% SSEA4 positive (figs. S5, G and I; S6, B and C; and S8, E and F). PRDM14-AID-VENUS naïve cells expressed NANOG, OCT4, and KLF4 (fig. S8D). Karyotype analysis of G-banded chromosomes (carried out by Cell Guidance Systems) confirmed that all cell lines have the normal complement of chromosomes, and representative karyotypes are shown in fig. S9A. Twenty cells were analyzed per cell line. For the FSPS13B CAGCAS9 B1 cell line, we examined the karyotype status of the cells that were used in the CRISPR screen by implementing a method called eSNP-Karyotyping (*64*). This approach enabled us to use the RNA-seq data collected from the samples at the time of the experiment to determine chromosome copy number. By adopting this approach, we examined the starting primed cells and the two cell populations that were flow-sorted for the CRISPR screen. The results show that all three samples have a normal chromosome copy number (fig. S9B).

#### Flow cytometry

Cells were dissociated using Accutase, washed, and passed through 50-μm cell strainers (VWR). Conjugated antibodies and Fixable Viability Dye-eF780 (eBioscience) were mixed with 50 μl of Brilliant stain buffer (BD Biosciences) and applied to 50 μl of cells (500,000 cells per reaction). Cells were incubated for 30 min at 4°C in the dark and washed twice with 2% fetal bovine serum (FBS) in PBS and centrifuged at 300*g* for 5 min. Cells were resuspended in 2% FBS in PBS and analyzed at the Babraham Institute Flow Core with a BD LSRFortessa cell analyzer (BD Biosciences) or a BD FACSAria Fusion for cell sorting. Single-stained cells or OneComp eBeads (eBioscience) were used for compensation calculations. Unstained cells and Fluorescence Minus One controls were used in cytometer and gating setup. A full gating strategy is shown in fig. S2A. Data were analyzed using FlowJo V10.1 software (BD Biosciences). The following fluorescent conjugated antibodies and flow cytometer laser and filter settings were used: CD24-BUV395 (1.25 μl per test; detected using the 355-nm laser with 379/28 filters), CD57-BV421 (2.5 μl per test; 405-nm laser with 450/40 filters), CD75-eF660 (2.5 μl per test; 640-nm laser with 670/14 filters), CD77-PE-CF594 (2.5 μl per test; 561-nm laser with 610/20 filters), Cd90.2-APC-Cy7 (2.5 μl per test; 640-nm laser with 780/60 filters), CD130-PE (10 μl per test; 561-nm laser with 585/15 filters), Fixable Viability Dye-eF780 (0.6 μl per test; 640-nm laser with 780/60 filters), SSEA4-BV605 (1.25 μl per test; 405-nm laser with 610/20 filters), and SUSD2-PE (0.5 μl per test; 561-nm laser with 585/15 filters).

#### Immunofluorescent microscopy

Cells were fixed for 15 min in 4% paraformaldehyde at room temperature and incubated in blocking and permeabilization solution (5% FBS and 0.1% Triton X-100 in PBS) for 1 hour at room temperature or overnight at 4°C. Cells were then incubated overnight in primary antibody at 4°C in blocking and permeabilization solution. After washing three times with blocking and permeabilization solution, cells were incubated with appropriate fluorescently conjugated secondary antibodies for 1 hour at room temperature. Cells were washed three times with PBS, with 4′,6-diamidino-2-phenylindole (0.5 μg/ml; DAPI) to stain DNA included in the second wash. Cells were imaged on either a NIKON A1-R confocal microscope with a 20× oil objective or a Zeiss Axio Observer with the Apotome 3 for structure illuminated optical sectioning, and *Z* stack images were processed with ImageJ. Antibody details are as follows. For primary antibodies, mouse anti-OCT4 (Santa Cruz Biotechnology, sc5279), goat anti-NANOG (R&D Systems, AF1997), rabbit anti-KLF17 (Atlas Antibodies, HPA024629), and goat anti-GFP (Abcam, ab6673) were all used at 1:300. Mouse anti-HDAC2 (Active Motif, 39533) and rabbit anti-TRIM28 (Abcam, ab10483) were used at 1:200. For secondary antibodies, Donkey anti-mouse IgG (H+L) Highly Cross-Adsorbed Secondary Antibody Alexa Fluor 647 (Invitrogen, A-31571), Donkey anti-rabbit IgG (H+L) Highly Cross-Adsorbed Secondary Antibody Alexa Fluor 555 (Invitrogen, A32794), and Donkey anti-goat IgG (H+L) Highly Cross-Adsorbed Secondary Antibody Alexa Fluor 488 (Invitrogen, A32814) were all used at 1:400. Donkey anti-rabbit IgG (H+L) Highly Cross-Adsorbed Secondary Antibody CF-568 (Sigma-Aldrich, SAB4600076) and Donkey anti-mouse IgG (H+L) Highly Cross-Adsorbed Secondary Antibody Alexa Fluor Plus 488 (Invitrogen, A32766) were used at 1:800.

#### RNA sequencing

RNA was extracted in TRIzol reagent (Thermo Fisher Scientific). Indexed libraries were constructed from 500 ng of total RNA using the NEBNext Ultra RNA Library Prep Kit for Illumina with the Poly(A) mRNA Magnetic Isolation Module (NEB). Library fragment size and concentration were determined using an Agilent Bioanalyzer 2100 and KAPA Library Quantification Kit (KAPA Biosystems). Samples were sequenced on an Illumina NextSeq 500 instrument as 75-bp single-end libraries at the Babraham Institute Sequencing Facility.

#### Quantitative, multiplexed rapid immunoprecipitation mass spectrometry of endogenous protein

Following established protocols (*42*, *43*), cells were dissociated with Accutase, washed with PBS, and cross-linked with 2 mM di(*N*-succinimidyl) glutarate (Sigma-Aldrich) in PBS for 45 min at room temperature with shaking and then with 1% methanol-free paraformaldehyde (Agar Scientific) for 12.5 min at room temperature with shaking. Fixation was quenched with 0.125 M glycine for 5 min at room temperature with shaking. Cells were washed with PBS containing cOmplete EDTA-free protease inhibitors (Roche), and MEFs were depleted using MACS columns, as described above. Twenty million PSCs per RIME were resuspended in 10 ml of nuclei extraction buffer [10 mM Hepes (pH 7.5), 10 mM EDTA (pH 8.0), 0.5 mM EGTA, and 0.75% Triton X-100] and incubated for 10 min at 4°C with rotation. After centrifugation, nuclei were resuspended in 10 ml of nuclei wash buffer [10 mM Hepes (pH 7.5), 200 mM NaCl, 1 mM EDTA (pH 8.0), and 0.5 mM EGTA] and incubated for 10 min at 4°C with rotation. Cells were pelleted and resuspended in 600 μl of lysis and sonication buffer [25 mM tris (pH 7.5), 150 mM NaCl, 5 mM EDTA (pH 8.0), 0.1% Triton X-100, 1% SDS, and 0.5% sodium deoxycholate], transferred to Protein LoBind tubes, and incubated for 30 min at 4°C to allow cell lysis. Chromatin was fragmented by sonication on a Microson ultrasonic cell disruptor XL Misonix wand sonicator with output setting of 10 W using 30, 32, or 34 sonication cycles (15 s on and 30 s off) for primed, 48-hour 5i/L/A and naïve PSC chromatin, respectively. Triton X-100 was added to the fragmented chromatin to a final 1% before immunoprecipitation. Magnetic Protein A Dynabeads (Thermo Fisher Scientific), 100 μl per immunoprecipitation, were washed three times with cold 0.5% BSA/PBS, and 5 μg of anti-PCGF3+5 antibody (Abcam, ab201510) was immobilized on the Protein A beads in 250 μl of cold PBS/0.5% BSA for at least 5 hours at 4°C with rotation. The three-wash steps with 0.5% BSA/PBS were then repeated, and the fragmented chromatin was added to the beads overnight at 4°C with rotation. The next day, the protein of interest that was bound to the magnetic beads was washed stringently 10 times with RIME radioimmunoprecipitation assay (RIPA) buffer [50 mM Hepes (pH 7.5), 500 mM LiCl 500, 1 mM EDTA (pH 8.0), 1% NP-40, and 0.7% sodium deoxycholate] and twice with 100 mM ammonium bicarbonate. All visible liquid was removed from the beads, which were then frozen at −80°C and taken to the Cancer Research UK Cambridge Proteomics Facility for on-bead trypsin digestion, sample processing, multiplexing, MS, and preliminary analysis. Samples were prepared as described previously (*43*). Briefly, after on-bead tryptic digestion, C18 cleaned peptides were labeled with the TMT-16plex reagents (Thermo Fisher Scientific) for 1 hour. Samples were mixed and fractionated with reversed-phase cartridges at high pH (Pierce). Nine fractions were collected using different elution solutions in the range of 5 to 50% ACN (Acetonitrile). Peptide fractions were reconstituted in 0.1% formic acid and analyzed on a Dionex Ultimate 3000 ultrahigh-performance liquid chromatography system coupled with the nano-ESI Fusion Lumos mass spectrometer (Thermo Fisher Scientific). Samples were loaded on the Acclaim PepMap 100, 100 μm by 2 cm C18, 5 μm, 100-Å trapping column with the ulPickUp injection method using the loading pump at a flow rate of 5 μl/min for 10 min. For the peptide separation, the EASY-Spray analytical column 75 μm by 25 cm, C18, 2 μm, 100-Å column was used for multistep gradient elution at a flow rate of 300 nl/min. Mobile phase A was composed of 2% acetonitrile and 0.1% formic acid, and mobile phase B was composed of 80% acetonitrile and 0.1% formic acid. Peptides were eluted using a gradient as follows: 0 to 10 min, 5% mobile phase B; 10 to 90 min, 5 to 38% mobile phase B; 90 to 100 min, 38 to 95% B; 100 to 105 min, 95% B; 105 to 110 min, 95 to 5% B; and 110 to 120 min, 5% B. Data-dependent acquisition began with an MS survey scan in the Orbitrap [380 to 1500 mass/charge ratio (*m*/*z*), resolution of 120,000 full width at half maximum (FWHM), automatic gain control (AGC) target of 3 × 10^5^, and maximum injection time of 100 ms]. MS2 analysis consisted of collision-induced dissociation (CID), quadrupole ion trap analysis, AGC target of 1 × 10^4^, normalized collision energy of 32, *q* value of 0.25, maximum injection time of 50 ms, an isolation window at 0.7, and a dynamic exclusion duration of 45 s. MS2-MS3 was conducted using sequential precursor selection methodology with the top10 setting. Higher energy collision dissociation (HCD)–MS3 analysis was performed with MS2 isolation window 2.0 Th. The HCD collision energy was set at 50%, and the detection was performed with Orbitrap resolution of 50,000 FWHM and in the scan range of 100 to 400 *m*/*z*. AGC target was 1 × 10^5^, with the maximum injection time of 105 ms.

#### ChIP sequencing

Cells were harvested, fixed, lysed, and sonicated as described above for qPLEX-RIME except that the MACS fibroblast depletion step was omitted. Fragmented chromatin was centrifuged at 20,000*g* for 15 min at 4°C, and the 600-μl supernatant was retained and diluted 1:10 with ChIP dilution buffer [150 mM NaCl, 25 mM tris (pH 7.5), 5 mM EDTA, 1% Triton X-100, 0.1% SDS, and 0.5% sodium deoxycholate] to 6-ml total volume and split across six Protein LoBind tubes. Five percent of the diluted chromatin was taken as an input for each condition, and the remaining diluted supernatant was incubated with a total of 10 μg of antibody overnight at 4°C. Antibodies were PCGF3+5 (Abcam, ab201510) and RING1B (*65*). Magnetic Protein A or G Dynabeads (Thermo Fisher Scientific), 60 μl per immunoprecipitation, were washed three times with 1 ml of cold wash buffer A [50 mM tris (pH 8), 150 mM NaCl, 0.1% SDS, 0.5% sodium deoxycholate, 1% NP-40, and 1 mM EDTA] and then blocked for 1 hour with 4 μl of yeast tRNA (10 mg/ml) (Thermo Fisher Scientific) and 10 μl of BSA (10 mg/ml) (NEB) in 1 ml of cold wash buffer A at 4°C with rotation. Blocked beads were washed three times with 1 ml of wash buffer A, and the 60-μl initial Protein A/G Dynabeads volume was made up to 200-μl volume with wash buffer A and incubated overnight at 4°C with rotation. Protein A/G beads (33.3 μl) were added to each of the six tubes containing antibody-bound chromatin, which was then incubated for at least 7 hours at 4°C with rotation to immobilize antibody-bound chromatin on the beads. The magnetic beads bound to antibody-chromatin complexes were rinsed once with wash buffer A and then washed twice for 10 min at 4°C with rotation with 1 ml of wash buffer A, and the beads split across six tubes were pooled into a single Protein LoBind tube across these two washes. The beads were washed once with 1 ml of wash buffer B [50 mM tris (pH 8.0), 500 mM NaCl, 0.1% SDS, 0.5% sodium deoxycholate, 1% NP-40, and 1 mM EDTA] and once with 1 ml of wash buffer C [50 mM tris (pH 8.0), 250 mM LiCl, 0.5% sodium deoxycholate, 1% NP-40, and 1 mM EDTA] and rinsed with 1× TE buffer. Chromatin was eluted off the beads in 450 μl of elution buffer (1% SDS and 0.1 M NaHCO_3_) containing 11 μl of proteinase K (20 mg/ml), and 5 μl ribonuclease A (10 mg/ml) (Promega) was added, including to the input samples, and incubated at 37°C for 2 hours, followed by an overnight incubation at 65°C with shaking to reverse cross-link protein from DNA. DNA was purified using AMPure XP beads (Beckman Coulter) into DNA LoBind tubes (Eppendorf) and eluted in 50 μl of buffer EB. DNA was quantified using a Qubit fluorometer double-stranded DNA high-sensitivity assay kit (Thermo Fisher Scientific), and libraries were prepared using a NEBNext Ultra II DNA library preparation kit for Illumina (NEB) using the manufacturer’s protocol, with libraries indexed using NEBNext Multiplex Oligos for Illumina (Index Primers Set 1 and Set 2) (NEB). Following library preparation, library fragment size and concentration were determined using a Qubit fluorometer double-stranded DNA high-sensitivity assay kit, using an Agilent Bioanalyzer 2100, and the KAPA Library Quantification Kit (KAPA Biosystems). Samples were sequenced on an Illumina NextSeq500 instrument as high-output 75-bp single-end reads at the Babraham Institute Next Generation Sequencing Facility. Two independent biological replicates were prepared for each ChIP in each cell type.

#### Western blotting

Whole-cell lysates were extracted in RIPA buffer [150 mM NaCl, 1% NP-40, 0.5% sodium deoxycholate, 0.1% SDS, and 50 mM tris (pH 8.0)] containing 1× cOmplete EDTA-free protease inhibitors. Protein concentration was determined by Bradford assay. Proteins were denatured by boiling at 95°C in 5× protein loading dye [4% SDS, 0.25 M tris (pH 6.8), 1 μM bromophenol blue, 0.5 mM dithiothreitol (DTT), and 30% glycerol] for 5 min. Protein was separated by electrophoresis on a 12% SDS–polyacrylamide gel alongside a prestained protein standard (Bio-Rad) to assess protein molecular weights. Protein was transferred onto a methanol-activated 0.45-μm Immobilon-P polyvinylidene difluoride membrane at 15 V for 1 hour. Membranes were blocked for 3 hours at room temperature in TBS-T (1× tris-buffered saline and 0.05% Tween 20) containing 5% dried skimmed milk and were hybridized overnight at 4°C with primary antibody diluted in TBS-T 5% milk. Membranes were washed for 10 min, three times with TBS-T before incubation with appropriate horseradish peroxidase (HRP)– or fluorophore-conjugated secondary antibody diluted in TBS-T 5% milk/BSA for 1 hour at room temperature. Membranes were washed for 10 min, three times with TBS-T and then once with 1× TBS before detection using either Odyssey Imaging Systems (LI-COR Biosciences) or ECL Prime Western Blotting detection reagent (Amersham Biosciences). Primary antibody details are as follows: PCGF3+5 (1:500; Abcam, ab201510), RING1B (1:1000) (*65*), AUTS2 (1:500; Abcam, ab96326), FBRS (1:500; Abcam, ab264404), RYBP (1:1000; Abcam, ab185971), PRDM14 (1:500; Abcam, ab187881), β-actin (1:1000; Sigma-Aldrich, A5441), and tubulin (Sigma-Aldrich, T6199). Secondary antibody details are as follows: anti-mouse IgG (H+L)-HRP (1:10,000; Bio-Rad, 1706516), anti-rabbit IgG (H+L)-HRP (1:10,000; Bio-Rad, 1706515), anti-mouse IgG (H+L)–DyLight-680 (1:10,000; Invitrogen, SA5-10170), and anti-rabbit IgG (H+L)–DyLight-800 (1:10,000; Invitrogen, SA5-10044).

#### Coimmunoprecipitation

Cells were washed with cold PBS and harvested by the addition of 500 μl of ice-cold Co-IP buffer 1 [10 mM Hepes (pH 7.5), 1.5 mM MgCl_2_, 10 mM KCl, 0.5 mM DTT, 0.05% NP-40, Benzonase (250 U/μl), and 1× cOmplete EDTA-free protease inhibitor] directly onto 15-cm tissue culture plates and by scraping. The cell suspension was transferred to tubes and incubated on ice for 10 min. Cells were collected by centrifugation at 1000*g* for 10 min at 4°C, and the nuclear pellet was resuspended in 376 μl of ice-cold Co-IP buffer 2 [5 mM Hepes (pH 7.9), 1.5 mM MgCl_2_, 0.2 mM EDTA, 0.5 mM DTT, 26% (v/v) glycerol, Benzonase (250 U/μl), and 1× cOmplete EDTA-free protease inhibitor], supplemented with 300 mM NaCl to lyse nuclear membranes. Nuclei were further lysed with 20 strokes with a Dounce homogenizer and incubated on ice for 30 min. Lysates were centrifuged at 24,000*g* for 20 min at 4°C; the supernatant was collected, and the protein was quantified by Bradford assay. For immunoprecipitation, 50 μl of Protein A or G Dynabeads was washed three times with Co-IP wash buffer [10 mM tris-HCl (pH 7.5), 150 mM NaCl, and 0.5 mM EDTA]. RING1B (*65*) or PCGF3 antibody (10 μg) was immobilized onto the beads for 3 hours at 4°C. Antibody-immobilized beads were rewashed three times in Co-IP wash buffer to remove any unbound antibody. Nuclear protein (500 μg) was added to the antibody-immobilized beads, and 50 μg (10%) of the nuclear protein was set aside as an input. The immunoprecipitation was incubated overnight at 4°C with rotation. The beads were washed three times in Co-IP wash buffer, and the immunocomplexes were eluted in 5× protein loading dye by boiling at 75°C for 10 min. The inputs were prepared the same way. Immunoprecipitation samples and their corresponding inputs were analyzed by Western blotting.

### Statistical analysis

#### CRISPR-Cas9 screen

gRNA reads were first counted using an in-house program. Statistical analysis was then performed using MAGeCK (*33*) by comparing gRNA counts between (i) the day 21 cell population and the starting gRNA library plasmids for the primed proliferation screen and (ii) nascent naïve and refractory cell populations for the reprogramming screen. In the reprogramming screen, the gRNA counts for the two samples are compared to each other and not to the starting gRNA library. It is possible that some gRNAs will be enriched or depleted following expansion and selection; for example, genes essential for the growth of primed PSCs may be lost before reprogramming is initiated. This might eliminate genes that are broadly essential for cell growth, thereby allowing us to focus on identifying genes that are implicated in naïve cell reprogramming. Similarly, the experimental design is unaffected if any gRNAs are enriched in the starting cell population following expansion and selection, as these genes would still be found as “hits” if their relative abundance differed between the reprogrammed and not reprogrammed samples.

#### Naïve colony-forming assay

A one-sided Student’s *t* test was used to compare nascent naïve colonies with refractory populations after cell sorting.

#### Flow cytometry

WT, *PCGF3* heterozygous, and *PCGF3* rescue lines were compared to both of the *PCGF3* KO lines using a one-way analysis of variance (ANOVA) with Tukey correction. A two-tailed *t* test was used to calculate the *P* value for the difference in the proportion of reprogrammed cells within the population with HDAC inhibitor treatments.

#### RNA-seq analysis

RNA-seq reads were trimmed using trim galore v0.6.5 (www.bioinformatics.babraham.ac.uk/projects/trim_galore/) using default parameters to remove the standard Illumina adapter sequence. Reads were mapped to the human GRCh38 genome assembly using HISAT 2.1.0 guided by the gene models from the Ensembl v70 release. SAMtools was used to convert to BAM files that were imported to SeqMonk v45.0 (www.bioinformatics.babraham.ac.uk/projects/seqmonk/). Raw read counts per transcript were calculated using the RNA-seq quantitation pipeline on the Ensembl v70 gene set using directional counts. Data were analyzed using DESeq and principal components analysis implemented in SeqMonk. Differentially expressed genes were identified using DESeq2, using a Wald test and Benjamini-Hochberg correction with a false discovery rate (FDR) of <0.05. *P* values for comparison of fold change in expression of PRC1.3 ChIP targets were calculated using a Kruskal-Wallis test with Dunn’s multiple comparisons test. Published RNA-seq datasets (*10*, *32*, *47*, *66*–*69*) were analyzed in R or SeqMonk.

#### ChIP-seq analysis

Reads were trimmed using trim galore (www.bioinformatics.babraham.ac.uk/projects/trim_galore/) using default parameters to remove the standard Illumina adapter sequence and mapped to the human genome GRCh38 using Bowtie2. BAM files were imported to SeqMonk (www.bioinformatics.babraham.ac.uk/projects/seqmonk/), and reads were extended by 200 bp at their 5′ end to approximate the true insert size. Regions of coverage outliers were excluded. To identify PRC1.3 target promoters, 4-kb probes were generated centered on annotated transcriptional start sites. Nonduplicated reads were quantified and corrected per million mapped reads. Probes with log_2_ RPM (Reads Per Million) > 3 in the RING1B ChIP samples and log_2_ RPM > 1 in the PCGF3 ChIP samples were retained. Quantitation values were matched normalized across the RING1B ChIP datasets and were percentile normalized (75%) across the PCGF3 ChIP datasets. Probes in the PCGF3 ChIP samples were retained if they had a *P* value of <0.05 (after multiple testing correction) based on a Limma test between WT and PCGF3 KO samples. Retained RING1B and PCGF3 probes were overlapped to identify regions of PRC1.3 occupancy. Probes were name-matched to genes and deduplicated by name. ChIP-seq peaks were called using a MACS implementation in SeqMonk. Each ChIP replicate was analyzed separately using parameters *q* < 10^−12^ for RING1B and *q* < 10^−5^ for PCGF3 and with sonicated fragment size of 300. Peaks identified in both replicates were retained and were filtered by signal intensity, retaining only peaks that overlap with at least one 500-bp window in which log_2_ RPM > 0. PCGF3 peaks were removed if they overlapped with peaks in *PCGF3* KO samples (*n* = 20 peaks in primed cells and *n* = 4 peaks in naïve cells). Retained RING1B and PCGF3 peaks were overlapped to identify regions of PRC1.3 occupancy. Matched regions with RING1B occupancy but not PCGF3 occupancy were identified by filtering retained RING1B peaks that were further than 10 kb from a PCGF3 peak. The control peaks were filtered to select a set of regions with a similar distribution of RING1B ChIP quantified values compared to the PRC1.3 peak set. PRDM14 ChIP signal was compared between PCGF3- and RING1B-bound sites versus matched RING1B-only sites using a two-sided Mann-Whitney test.

Heatmaps were generated as follows. BAM files were sorted and indexed using SAMtools. Sorted indexed BAMs were converted to bigWig format using deepTools bamCoverage with the following parameters: --binSize 10 --normalizeUsing RPGC --effectiveGenomeSize 2913022398 --extendReads 200 --ignoreForNormalization ChrM ChrY --minMappingQuality 20. To generate coverage tracks for analysis over regions of interest, input files were subtracted from samples using bigwigCompare with the following parameter: --operation subtract. For visualization, input-subtracted replicates were merged using bigwigCompare with the following parameter: --operation mean. To generate aligned probe plots, first, computeMatrix was run on each input-normalized coverage file using the following parameters: --upstream 10000 --downstream 10000 --missingDataAsZero —skipZeros. Plots were then generated using plotHeatmap with interpolationMethod set to bilinear, and samples were sorted by PCGF3 signal.

#### Analysis of protein compartmentalization

Protein localization information was retrieved from the COMPARTMENTS database from the Jensen Laboratory in json format using a REST API client (*70*). Within these files, confidence in the location of individual proteins is assigned a score based on experimental validation, knowledge, or various computational prediction methods. For proteins annotated within multiple subcellular compartments, the highest score was used. Plots were produced using R using custom scripts. Statistical significance of enrichment over a “background” gene set (all genes in the screening library) was carried out using a hypergeometric test. Adjusted *P* values were calculated using Fisher’s exact test.

#### Gene ontology analysis

Gene ontology (GO) information was accessed through the R package enrichR (*71*) for the following GO terms: GO_Cellular_Component_2018, GO_Molecular_Function_2018, and GO_Biological_Process_2018. GO terms were ranked by significance, and −log_10_-adjusted *P* values were plotted using R. Adjusted *P* values were calculated using Fisher’s exact test.

#### Motif analysis

Motif analysis was performed using AME (*72*) on the central 500-bp region within naïve PRC1.3 peaks. Regions matched for GC content were generated using custom Python scripts. The motif database used was HOCOMOCO v11 core human mono meme format. FIMO (*73*) was used to identify the number of PRDM14 motifs in PRC1.3 peaks versus matched RING1B-only peaks or versus regions matched for GC content. Over half of all naïve PRC1.3 peaks (trimmed to the central 500 bp) contained at least one PRDM14 motif (53%; 201 of 378) compared to ~6% in the RING1B-only peak set (6.4%; 19 of 295) and to ~5% in the GC-matched set (5.3%; 20 of 378). These values were compared using a two-sided Fisher’s exact test. The significance of the enrichment of PRDM14 motifs in naïve PRC1.3 peaks compared to a control subset was compared using a one-sided Fisher’s exact test with Bonferroni correction.

#### Gene set enrichment analysis

Gene set enrichment analysis (GSEA) was calculated using the GSEAPreranked tool within GSEA software (www.broadinstitute.org/gsea). Input data were a ranked gene list ordered by fold change expression between IAA-treated and untreated PRDM14-VENUS-AID PSCs [*n* = 35721; (*47*)] and a set of PRC1.3 targets (*n* = 267; defined by high RING1B and PCGF3 promoter-localized ChIP-seq values in PSCs). Default settings were used with 1000 gene set permutations. The positive enrichment score is calculated using a Kolmogorov-Smirnov test with FDR < 0.001.

#### qPLEX-RIME analysis

The Proteome Discoverer 2.1 (Thermo Fisher Scientific) was used for the processing of CID tandem mass spectra. The SequestHT search engine was used, and all the spectra were searched against the UniProt *Homo sapiens* FASTA database (taxon ID 9606, version January 2020). All searches were performed using a static modification TMT pro 16plex (+304.207 Da) at any N terminus and on lysine. Methionine oxidation (+15.9949 Da) and deamidation on asparagine and glutamine (+0.984) were included as dynamic modifications. Mass spectra were searched using precursor ion tolerance of 20 parts per million and fragment ion tolerance of 0.5 Da. For peptide confidence, 1% FDR was applied, and peptides uniquely matched to a protein were used for quantification.

Data processing, normalization, and statistical analysis were carried out using the qPLEXanalyzer (*43*) package from Bioconductor. Peptide intensities were normalized using median scaling, and protein level quantification was obtained by the summation of the normalized peptide intensities. A statistical analysis of differentially regulated proteins was carried out using the Limma method. Multiple testing correction of *P* values was applied using the Benjamini-Hochberg method to control the FDR.

## Notes

View/request a protocol for this paper from *Bio-protocol*.

## References

[1] J. Rossant, P. P. L. Tam, New insights into early human development: Lessons for stem cell derivation and differentiation. Cell Stem Cell 20, 18–28 (2017). 28061351 10.1016/j.stem.2016.12.004

[2] V. L. Mascetti, R. A. Pedersen, Human-mouse chimerism validates human stem cell pluripotency. Cell Stem Cell 18, 67–72 (2016). 26712580 10.1016/j.stem.2015.11.017 PMC4712187

[3] J. A. Thomson, Embryonic stem cell lines derived from human blastocysts. Science 282, 1145–1147 (1998). 9804556 10.1126/science.282.5391.1145

[4] J. Wu, D. Okamura, M. Li, K. Suzuki, C. Luo, L. Ma, Y. He, Z. Li, C. Benner, I. Tamura, M. N. Krause, J. R. Nery, T. Du, Z. Zhang, T. Hishida, Y. Takahashi, E. Aizawa, N. Y. Kim, J. Lajara, P. Guillen, J. M. Campistol, C. R. Esteban, P. J. Ross, A. Saghatelian, B. Ren, J. R. Ecker, J. C. Izpisua Belmonte, An alternative pluripotent state confers interspecies chimaeric competency. Nature 521, 316–321 (2015). 25945737 10.1038/nature14413 PMC5278765

[5] B. E. Reubinoff, M. F. Pera, C. Y. Fong, A. Trounson, A. Bongso, Embryonic stem cell lines from human blastocysts: Somatic differentiation in vitro. Nat. Biotechnol. 18, 399–404 (2000). 10748519 10.1038/74447

[6] K. Takahashi, K. Tanabe, M. Ohnuki, M. Narita, T. Ichisaka, K. Tomoda, S. Yamanaka, Induction of pluripotent stem cells from adult human fibroblasts by defined factors. Cell 131, 861–872 (2007). 18035408 10.1016/j.cell.2007.11.019

[7] J. Yu, M. A. Vodyanik, K. Smuga-Otto, J. Antosiewicz-Bourget, J. L. Frane, S. Tian, J. Nie, G. A. Jonsdottir, V. Ruotti, R. Stewart, I. I. Slukvin, J. A. Thomson, Induced pluripotent stem cell lines derived from human somatic cells. Science 318, 1917–1920 (2007). 18029452 10.1126/science.1151526

[8] L. Weinberger, M. Ayyash, N. Novershtern, J. H. Hanna, Dynamic stem cell states: Naive to primed pluripotency in rodents and humans. Nat. Rev. Mol. Cell Biol. 17, 155–169 (2016). 26860365 10.1038/nrm.2015.28

[9] K. C. Davidson, E. A. Mason, M. F. Pera, The pluripotent state in mouse and human. Development 142, 3090–3099 (2015). 26395138 10.1242/dev.116061

[10] T. Nakamura, I. Okamoto, K. Sasaki, Y. Yabuta, C. Iwatani, H. Tsuchiya, Y. Seita, S. Nakamura, T. Yamamoto, M. Saitou, A developmental coordinate of pluripotency among mice, monkeys and humans. Nature 537, 57–62 (2016). 27556940 10.1038/nature19096

[11] A. Sahakyan, R. Kim, C. Chronis, S. Sabri, G. Bonora, T. W. Theunissen, E. Kuoy, J. Langerman, A. T. Clark, R. Jaenisch, K. Plath, Human naive pluripotent stem cells model X chromosome dampening and x inactivation. Cell Stem Cell 20, 87–101 (2017). 27989770 10.1016/j.stem.2016.10.006 PMC5218861

[12] C. Vallot, C. Patrat, A. J. Collier, C. Huret, M. Casanova, T. M. Liyakat Ali, M. Tosolini, N. Frydman, E. Heard, P. J. Rugg-Gunn, C. Rougeulle, XACT noncoding RNA competes with XIST in the control of X chromosome activity during human early development. Cell Stem Cell 20, 102–111 (2017). 27989768 10.1016/j.stem.2016.10.014 PMC5222720

[13] G. G. Stirparo, T. Boroviak, G. Guo, J. Nichols, A. Smith, P. Bertone, Integrated analysis of single-cell embryo data yields a unified transcriptome signature for the human pre-implantation epiblast. Development 145, dev158501 (2018). 29361568 10.1242/dev.158501 PMC5818005

[14] K. Huang, T. Maruyama, G. Fan, The naive state of human pluripotent stem cells: A synthesis of stem cell and preimplantation embryo transcriptome analyses. Cell Stem Cell 15, 410–415 (2014). 25280217 10.1016/j.stem.2014.09.014 PMC5507179

[15] W. A. Pastor, D. Chen, W. Liu, R. Kim, A. Sahakyan, A. Lukianchikov, K. Plath, S. E. Jacobsen, A. T. Clark, Naive human pluripotent cells feature a methylation landscape devoid of blastocyst or germline memory. Cell Stem Cell 18, 323–329 (2016). 26853856 10.1016/j.stem.2016.01.019 PMC4779431

[16] H. Guo, P. Zhu, L. Yan, R. Li, B. Hu, Y. Lian, J. Yan, X. Ren, S. Lin, J. Li, X. Jin, X. Shi, P. Liu, X. Wang, W. Wang, Y. Wei, X. Li, F. Guo, X. Wu, X. Fan, J. Yong, L. Wen, S. X. Xie, F. Tang, J. Qiao, The DNA methylation landscape of human early embryos. Nature 511, 606–610 (2014). 25079557 10.1038/nature13544

[17] C. Dong, M. Beltcheva, P. Gontarz, B. Zhang, P. Popli, L. A. Fischer, S. A. Khan, K.-M. Park, E.-J. Yoon, X. Xing, R. Kommagani, T. Wang, L. Solnica-Krezel, T. W. Theunissen, Derivation of trophoblast stem cells from naïve human pluripotent stem cells. eLife 9, e52504 (2020). 32048992 10.7554/eLife.52504 PMC7062471

[18] J. K. Cinkornpumin, S. Y. Kwon, Y. Guo, I. Hossain, J. Sirois, C. S. Russett, H.-W. Tseng, H. Okae, T. Arima, T. F. Duchaine, W. Liu, W. A. Pastor, Naive human embryonic stem cells can give rise to cells with a trophoblast-like transcriptome and methylome. Stem Cell Reports 15, 198–213 (2020). 32619492 10.1016/j.stemcr.2020.06.003 PMC7363941

[19] M. Linneberg-Agerholm, Y. F. Wong, J. A. Romero Herrera, R. S. Monteiro, K. G. V. Anderson, J. M. Brickman, Naïve human pluripotent stem cells respond to Wnt, nodal and LIF signalling to produce expandable naïve extra-embryonic endoderm. Development 146, dev180620 (2019). 31740534 10.1242/dev.180620

[20] G. Guo, G. G. Stirparo, S. Strawbridge, D. Spindlow, J. Yang, J. Clarke, A. Dattani, A. Yanagida, M. A. Li, S. Myers, B. N. Özel, J. Nichols, A. Smith, Human naïve epiblast cells possess unrestricted lineage potential. Cell Stem Cell 28, 1040–1056.e6 (2021). 33831366 10.1016/j.stem.2021.02.025 PMC8189439

[21] G. Castel, D. Meistermann, B. Bretin, J. Firmin, J. Blin, S. Loubersac, A. Bruneau, S. Chevolleau, S. Kilens, C. Chariau, A. Gaignerie, Q. Francheteau, H. Kagawa, E. Charpentier, L. Flippe, V. François-Campion, S. Haider, B. Dietrich, M. Knöfler, T. Arima, J. Bourdon, N. Rivron, D. Masson, T. Fournier, H. Okae, T. Fréour, L. David, Induction of human trophoblast stem cells from somatic cells and pluripotent stem cells. Cell Rep. 33, 108419 (2020). 33238118 10.1016/j.celrep.2020.108419

[22] A. Yanagida, D. Spindlow, J. Nichols, A. Dattani, A. Smith, G. Guo, Naive stem cell blastocyst model captures human embryo lineage segregation. Cell Stem Cell 28, 1016–1022.e4 (2021). 33957081 10.1016/j.stem.2021.04.031 PMC8189436

[23] X. Liu, J. P. Tan, J. Schröder, A. Aberkane, J. F. Ouyang, M. Mohenska, S. M. Lim, Y. B. Y. Sun, J. Chen, G. Sun, Y. Zhou, D. Poppe, R. Lister, A. T. Clark, O. J. L. Rackham, J. Zenker, J. M. Polo, Modelling human blastocysts by reprogramming fibroblasts into iBlastoids. Nature 591, 627–632 (2021). 33731926 10.1038/s41586-021-03372-y

[24] L. Yu, Y. Wei, J. Duan, D. A. Schmitz, M. Sakurai, L. Wang, K. Wang, S. Zhao, G. C. Hon, J. Wu, Blastocyst-like structures generated from human pluripotent stem cells. Nature 591, 620–626 (2021). 33731924 10.1038/s41586-021-03356-y

[25] Y. Takashima, G. Guo, R. Loos, J. Nichols, G. Ficz, F. Krueger, D. Oxley, F. Santos, J. Clarke, W. Mansfield, W. Reik, P. Bertone, A. Smith, Resetting transcription factor control circuitry toward ground-state pluripotency in human. Cell 158, 1254–1269 (2014). 25215486 10.1016/j.cell.2014.08.029 PMC4162745

[26] T. W. Theunissen, B. E. Powell, H. Wang, M. Mitalipova, D. A. Faddah, J. Reddy, Z. P. Fan, D. Maetzel, K. Ganz, L. Shi, T. Lungjangwa, S. Imsoonthornruksa, Y. Stelzer, S. Rangarajan, A. D’Alessio, J. Zhang, Q. Gao, M. M. Dawlaty, R. A. Young, N. S. Gray, R. Jaenisch, Systematic identification of culture conditions for induction and maintenance of naive human pluripotency. Cell Stem Cell 15, 471–487 (2014). 25090446 10.1016/j.stem.2014.07.002 PMC4184977

[27] G. Guo, F. von Meyenn, F. Santos, Y. Chen, W. Reik, P. Bertone, A. Smith, J. Nichols, Naive pluripotent stem cells derived directly from isolated cells of the human inner cell mass. Stem Cell Reports 6, 437–446 (2016). 26947977 10.1016/j.stemcr.2016.02.005 PMC4834040

[28] X. Liu, C. M. Nefzger, F. J. Rossello, J. Chen, A. S. Knaupp, J. Firas, E. Ford, J. Pflueger, J. M. Paynter, H. S. Chy, C. M. O’Brien, C. Huang, K. Mishra, M. Hodgson-Garms, N. Jansz, S. M. Williams, M. E. Blewitt, S. K. Nilsson, R. B. Schittenhelm, A. L. Laslett, R. Lister, J. M. Polo, Comprehensive characterization of distinct states of human naive pluripotency generated by reprogramming. Nat. Methods 14, 1055–1062 (2017). 28945704 10.1038/nmeth.4436

[29] X. Liu, J. F. Ouyang, F. J. Rossello, J. P. Tan, K. C. Davidson, D. S. Valdes, J. Schröder, Y. B. Y. Sun, J. Chen, A. S. Knaupp, G. Sun, H. S. Chy, Z. Huang, J. Pflueger, J. Firas, V. Tano, S. Buckberry, J. M. Paynter, M. R. Larcombe, D. Poppe, X. Y. Choo, C. M. O’Brien, W. A. Pastor, D. Chen, A. L. Leichter, H. Naeem, P. Tripathi, P. P. Das, A. Grubman, D. R. Powell, A. L. Laslett, L. David, S. K. Nilsson, A. T. Clark, R. Lister, C. M. Nefzger, L. G. Martelotto, O. J. L. Rackham, J. M. Polo, Reprogramming roadmap reveals route to human induced trophoblast stem cells. Nature 586, 101–107 (2020). 32939092 10.1038/s41586-020-2734-6

[30] G. Guo, F. von Meyenn, M. Rostovskaya, J. Clarke, S. Dietmann, D. Baker, A. Sahakyan, S. Myers, P. Bertone, W. Reik, K. Plath, A. Smith, Epigenetic resetting of human pluripotency. Development 144, 2748–2763 (2017). 28765214 10.1242/dev.146811 PMC5560041

[31] J. Bayerl, M. Ayyash, T. Shani, Y. S. Manor, O. Gafni, R. Massarwa, Y. Kalma, A. Aguilera-Castrejon, M. Zerbib, H. Amir, D. Sheban, S. Geula, N. Mor, L. Weinberger, S. Naveh Tassa, V. Krupalnik, B. Oldak, N. Livnat, S. Tarazi, S. Tawil, E. Wildschutz, S. Ashouokhi, L. Lasman, V. Rotter, S. Hanna, D. Ben-Yosef, N. Novershtern, S. Viukov, J. H. Hanna, Principles of signaling pathway modulation for enhancing human naive pluripotency induction. Cell Stem Cell 28, 1549–1565.e12 (2021). 33915080 10.1016/j.stem.2021.04.001 PMC8423434

[32] A. J. Collier, S. P. Panula, J. P. Schell, P. Chovanec, A. Plaza Reyes, S. Petropoulos, A. E. Corcoran, R. Walker, I. Douagi, F. Lanner, P. J. Rugg-Gunn, Comprehensive cell surface protein profiling identifies specific markers of human naive and primed pluripotent states. Cell Stem Cell 20, 874–890.e7 (2017). 28343983 10.1016/j.stem.2017.02.014 PMC5459756

[33] W. Li, H. Xu, T. Xiao, L. Cong, M. I. Love, F. Zhang, R. A. Irizarry, J. S. Liu, M. Brown, X. S. Liu, MAGeCK enables robust identification of essential genes from genome-scale CRISPR/Cas9 knockout screens. Genome Biol. 15, 554 (2014). 25476604 10.1186/s13059-014-0554-4 PMC4290824

[34] C.-X. D. Toh, J.-W. Chan, Z.-S. Chong, H. F. Wang, H. C. Guo, S. Satapathy, D. Ma, G. Y. L. Goh, E. Khattar, L. Yang, V. Tergaonkar, Y.-T. Chang, J. J. Collins, G. Q. Daley, K. B. Wee, C. A. E. Farran, H. Li, Y.-P. Lim, F. A. Bard, Y.-H. Loh, RNAi reveals phase-specific global regulators of human somatic cell reprogramming. Cell Rep. 15, 2597–2607 (2016). 27292646 10.1016/j.celrep.2016.05.049

[35] N. P. Blackledge, R. J. Klose, The molecular principles of gene regulation by Polycomb repressive complexes. Nat. Rev. Mol. Cell Biol. 22, 815–833 (2021). 34400841 10.1038/s41580-021-00398-y PMC7612013

[36] D. Helmlinger, L. Tora, Sharing the SAGA. Trends Biochem. Sci. 42, 850–861 (2017). 28964624 10.1016/j.tibs.2017.09.001 PMC5660625

[37] L. Zimmerlin, T. S. Park, J. S. Huo, K. Verma, S. R. Pather, C. C. Talbot Jr., J. Agarwal, D. Steppan, Y. W. Zhang, M. Considine, H. Guo, X. Zhong, C. Gutierrez, L. Cope, M. V. Canto-Soler, A. D. Friedman, S. B. Baylin, E. T. Zambidis, Tankyrase inhibition promotes a stable human naïve pluripotent state with improved functionality. Development 143, 4368–4380 (2016). 27660325 10.1242/dev.138982 PMC5201042

[38] Z. Xu, A. M. Robitaille, J. D. Berndt, K. C. Davidson, K. A. Fischer, J. Mathieu, J. C. Potter, H. Ruohola-Baker, R. T. Moon, Wnt/β-catenin signaling promotes self-renewal and inhibits the primed state transition in naïve human embryonic stem cells. Proc. Natl. Acad. Sci. U.S.A. 113, E6382–E6390 (2016). 27698112 10.1073/pnas.1613849113 PMC5081574

[39] K. Wojdyla, A. J. Collier, C. Fabian, P. S. Nisi, L. Biggins, D. Oxley, P. J. Rugg-Gunn, Cell-surface proteomics identifies differences in signaling and adhesion protein expression between naive and primed human pluripotent stem cells. Stem Cell Reports 14, 972–988 (2020). 32302559 10.1016/j.stemcr.2020.03.017 PMC7220956

[40] N. Bredenkamp, G. G. Stirparo, J. Nichols, A. Smith, G. Guo, The cell-surface marker sushi containing domain 2 facilitates establishment of human naive pluripotent stem cells. Stem Cell Reports 12, 1212–1222 (2019). 31031191 10.1016/j.stemcr.2019.03.014 PMC6565611

[41] N. Shakiba, C. A. White, Y. Y. Lipsitz, A. Yachie-Kinoshita, P. D. Tonge, S. M. I. Hussein, M. C. Puri, J. Elbaz, J. Morrissey-Scoot, M. Li, J. Munoz, M. Benevento, I. M. Rogers, J. H. Hanna, A. J. R. Heck, B. Wollscheid, A. Nagy, P. W. Zandstra, CD24 tracks divergent pluripotent states in mouse and human cells. Nat. Commun. 6, 7329 (2015). 26076835 10.1038/ncomms8329 PMC4490408

[42] H. Mohammed, C. Taylor, G. D. Brown, E. K. Papachristou, J. S. Carroll, C. S. D’Santos, Rapid immunoprecipitation mass spectrometry of endogenous proteins (RIME) for analysis of chromatin complexes. Nat. Protoc. 11, 316–326 (2016). 26797456 10.1038/nprot.2016.020

[43] E. K. Papachristou, K. Kishore, A. N. Holding, K. Harvey, T. I. Roumeliotis, C. S. R. Chilamakuri, S. Omarjee, K. M. Chia, A. Swarbrick, E. Lim, F. Markowetz, M. Eldridge, R. Siersbaek, C. S. D’Santos, J. S. Carroll, A quantitative mass spectrometry-based approach to monitor the dynamics of endogenous chromatin-associated protein complexes. Nat. Commun. 9, 2311 (2018). 29899353 10.1038/s41467-018-04619-5 PMC5998130

[44] Z. Gao, J. Zhang, R. Bonasio, F. Strino, A. Sawai, F. Parisi, Y. Kluger, D. Reinberg, PCGF homologs, CBX proteins, and RYBP define functionally distinct PRC1 family complexes. Mol. Cell 45, 344–356 (2012). 22325352 10.1016/j.molcel.2012.01.002 PMC3293217

[45] S. Hauri, F. Comoglio, M. Seimiya, M. Gerstung, T. Glatter, K. Hansen, R. Aebersold, R. Paro, M. Gstaiger, C. Beisel, A high-density map for navigating the human polycomb complexome. Cell Rep. 17, 583–595 (2016). 27705803 10.1016/j.celrep.2016.08.096

[46] N.-Y. Chia, Y.-S. Chan, B. Feng, X. Lu, Y. L. Orlov, D. Moreau, P. Kumar, L. Yang, J. Jiang, M.-S. Lau, M. Huss, B.-S. Soh, P. Kraus, P. Li, T. Lufkin, B. Lim, N. D. Clarke, F. Bard, H.-H. Ng, A genome-wide RNAi screen reveals determinants of human embryonic stem cell identity. Nature 468, 316–320 (2010). 20953172 10.1038/nature09531

[47] A. Sybirna, W. W. C. Tang, M. Pierson Smela, S. Dietmann, W. H. Gruhn, R. Brosh, M. A. Surani, A critical role of PRDM14 in human primordial germ cell fate revealed by inducible degrons. Nat. Commun. 11, 1282 (2020). 32152282 10.1038/s41467-020-15042-0 PMC7062732

[48] D. Huangfu, R. Maehr, W. Guo, A. Eijkelenboom, M. Snitow, A. E. Chen, D. A. Melton, Induction of pluripotent stem cells by defined factors is greatly improved by small-molecule compounds. Nat. Biotechnol. 26, 795–797 (2008). 18568017 10.1038/nbt1418 PMC6334647

[49] P. Mali, B.-K. Chou, J. Yen, Z. Ye, J. Zou, S. Dowey, R. A. Brodsky, J. E. Ohm, W. Yu, S. B. Baylin, K. Yusa, A. Bradley, D. J. Meyers, C. Mukherjee, P. A. Cole, L. Cheng, Butyrate greatly enhances derivation of human induced pluripotent stem cells by promoting epigenetic remodeling and the expression of pluripotency-associated genes. Stem Cells 28, 713–720 (2010). 20201064 10.1002/stem.402 PMC3015217

[50] C. B. Ware, A. M. Nelson, B. Mecham, J. Hesson, W. Zhou, E. C. Jonlin, A. J. Jimenez-Caliani, X. Deng, C. Cavanaugh, S. Cook, P. J. Tesar, J. Okada, L. Margaretha, H. Sperber, M. Choi, C. A. Blau, P. M. Treuting, R. D. Hawkins, V. Cirulli, H. Ruohola-Baker, Derivation of naive human embryonic stem cells. Proc. Natl. Acad. Sci. U.S.A. 111, 4484–4489 (2014). 24623855 10.1073/pnas.1319738111 PMC3970494

[51] F. F. Wagner, Y.-L. Zhang, D. M. Fass, N. Joseph, J. P. Gale, M. Weïwer, P. McCarren, S. L. Fisher, T. Kaya, W.-N. Zhao, S. A. Reis, K. M. Hennig, M. Thomas, B. C. Lemercier, M. C. Lewis, J. S. Guan, M. P. Moyer, E. Scolnick, S. J. Haggarty, L.-H. Tsai, E. B. Holson, Kinetically selective inhibitors of histone deacetylase 2 (HDAC2) as cognition enhancers. Chem. Sci. 6, 804–815 (2015). 25642316 10.1039/c4sc02130d PMC4310013

[52] M. Yamaji, J. Ueda, K. Hayashi, H. Ohta, Y. Yabuta, K. Kurimoto, R. Nakato, Y. Yamada, K. Shirahige, M. Saitou, PRDM14 ensures naive pluripotency through dual regulation of signaling and epigenetic pathways in mouse embryonic stem cells. Cell Stem Cell 12, 368–382 (2013). 23333148 10.1016/j.stem.2012.12.012

[53] Z. Ma, T. Swigut, A. Valouev, A. Rada-Iglesias, J. Wysocka, Sequence-specific regulator Prdm14 safeguards mouse ESCs from entering extraembryonic endoderm fates. Nat. Struct. Mol. Biol. 18, 120–127 (2011). 21183938 10.1038/nsmb.2000

[54] A. Scelfo, D. Fernández-Pérez, S. Tamburri, M. Zanotti, E. Lavarone, M. Soldi, T. Bonaldi, K. J. Ferrari, D. Pasini, Functional landscape of PCGF proteins reveals both RING1A/B-dependent-and RING1A/B-independent-specific activities. Mol. Cell 74, 1037–1052.e7 (2019). 31029542 10.1016/j.molcel.2019.04.002 PMC6561742

[55] Z. Gao, P. Lee, J. M. Stafford, M. von Schimmelmann, A. Schaefer, D. Reinberg, An AUTS2-Polycomb complex activates gene expression in the CNS. Nature 516, 349–354 (2014). 25519132 10.1038/nature13921 PMC4323097

[56] N. A. Fursova, N. P. Blackledge, M. Nakayama, S. Ito, Y. Koseki, A. M. Farcas, H. W. King, H. Koseki, R. J. Klose, Synergy between variant PRC1 complexes defines polycomb-mediated gene repression. Mol. Cell 74, 1020–1036.e8 (2019). 31029541 10.1016/j.molcel.2019.03.024 PMC6561741

[57] M. Almeida, G. Pintacuda, O. Masui, Y. Koseki, M. Gdula, A. Cerase, D. Brown, A. Mould, C. Innocent, M. Nakayama, L. Schermelleh, T. B. Nesterova, H. Koseki, N. Brockdorff, PCGF3/5-PRC1 initiates Polycomb recruitment in X chromosome inactivation. Science 356, 1081–1084 (2017). 28596365 10.1126/science.aal2512 PMC6522364

[58] W. Zhao, Y. Huang, J. Zhang, M. Liu, H. Ji, C. Wang, N. Cao, C. Li, Y. Xia, Q. Jiang, J. Qin, Polycomb group RING finger proteins 3/5 activate transcription via an interaction with the pluripotency factor Tex10 in embryonic stem cells. J. Biol. Chem. 292, 21527–21537 (2017). 29054931 10.1074/jbc.M117.804054 PMC5766968

[59] G. Monderer-Rothkoff, N. Tal, M. Risman, O. Shani, M. Nissim-Rafinia, L. Malki-Feldman, V. Medvedeva, M. Groszer, E. Meshorer, S. Shifman, AUTS2 isoforms control neuronal differentiation. Mol. Psychiatry 26, 666–681 (2021). 30953002 10.1038/s41380-019-0409-1

[60] H. Rashidi, N.-T. Luu, S. M. Alwahsh, M. Ginai, S. Alhaque, H. Dong, R. A. Tomaz, B. Vernay, V. Vigneswara, J. M. Hallett, A. Chandrashekran, A. Dhawan, L. Vallier, M. Bradley, A. Callanan, S. J. Forbes, P. N. Newsome, D. C. Hay, 3D human liver tissue from pluripotent stem cells displays stable phenotype in vitro and supports compromised liver function in vivo. Arch. Toxicol. 92, 3117–3129 (2018). 30155720 10.1007/s00204-018-2280-2 PMC6132688

[61] K. Tzelepis, H. Koike-Yusa, E. De Braekeleer, Y. Li, E. Metzakopian, O. M. Dovey, A. Mupo, V. Grinkevich, M. Li, M. Mazan, M. Gozdecka, S. Ohnishi, J. Cooper, M. Patel, T. McKerrell, B. Chen, A. F. Domingues, P. Gallipoli, S. Teichmann, H. Ponstingl, U. McDermott, J. Saez-Rodriguez, B. J. P. Huntly, F. Iorio, C. Pina, G. S. Vassiliou, K. Yusa, A CRISPR dropout screen identifies genetic vulnerabilities and therapeutic targets in acute myeloid leukemia. Cell Rep. 17, 1193–1205 (2016). 27760321 10.1016/j.celrep.2016.09.079 PMC5081405

[62] S. H. Ong, Y. Li, H. Koike-Yusa, K. Yusa, Optimised metrics for CRISPR-KO screens with second-generation gRNA libraries. Sci. Rep. 7, 7384 (2017). 28785007 10.1038/s41598-017-07827-z PMC5547152

[63] H. Koike-Yusa, Y. Li, E.-P. Tan, M. D. C. Velasco-Herrera, K. Yusa, Genome-wide recessive genetic screening in mammalian cells with a lentiviral CRISPR-guide RNA library. Nat. Biotechnol. 32, 267–273 (2014). 24535568 10.1038/nbt.2800

[64] U. Weissbein, M. Schachter, D. Egli, N. Benvenisty, Analysis of chromosomal aberrations and recombination by allelic bias in RNA-seq. Nat. Commun. 7, 12144 (2016). 27385103 10.1038/ncomms12144 PMC4941052

[65] T. Atsuta, S. Fujimura, H. Moriya, M. Vidal, T. Akasaka, H. Koseki, Production of monoclonal antibodies against mammalian Ring1B proteins. Hybridoma 20, 43–46 (2001). 11289226 10.1089/027245701300060427

[66] T. Messmer, F. von Meyenn, A. Savino, F. Santos, H. Mohammed, A. T. L. Lun, J. C. Marioni, W. Reik, Transcriptional heterogeneity in naive and primed human pluripotent stem cells at single-cell resolution. Cell Rep. 26, 815, –824.e4 (2019). 10.1016/j.celrep.2018.12.099 PMC6344340 30673604

[67] M. Rostovskaya, G. G. Stirparo, A. Smith, Capacitation of human naïve pluripotent stem cells for multi-lineage differentiation. Development 146, dev172916 (2019). 30944104 10.1242/dev.172916 PMC6467473

[68] L. Xiang, Y. Yin, Y. Zheng, Y. Ma, Y. Li, Z. Zhao, J. Guo, Z. Ai, Y. Niu, K. Duan, J. He, S. Ren, D. Wu, Y. Bai, Z. Shang, X. Dai, W. Ji, T. Li, A developmental landscape of 3D-cultured human pre-gastrulation embryos. Nature 577, 537–542 (2020). 31830756 10.1038/s41586-019-1875-y

[69] S. Petropoulos, D. Edsgärd, B. Reinius, Q. Deng, S. P. Panula, S. Codeluppi, A. P. Reyes, S. Linnarsson, R. Sandberg, F. Lanner, Single-cell RNA-seq reveals lineage and X chromosome dynamics in human preimplantation embryos. Cell 165, 1012–1026 (2016). 27062923 10.1016/j.cell.2016.03.023 PMC4868821

[70] J. X. Binder, S. Pletscher-Frankild, K. Tsafou, C. Stolte, S. I. O’Donoghue, R. Schneider, L. J. Jensen, COMPARTMENTS: Unification and visualization of protein subcellular localization evidence. Database 2014, bau012 (2014). 24573882 10.1093/database/bau012 PMC3935310

[71] M. V. Kuleshov, M. R. Jones, A. D. Rouillard, N. F. Fernandez, Q. Duan, Z. Wang, S. Koplev, S. L. Jenkins, K. M. Jagodnik, A. Lachmann, M. G. McDermott, C. D. Monteiro, G. W. Gundersen, A. Ma’ayan, Enrichr: A comprehensive gene set enrichment analysis web server 2016 update. Nucleic Acids Res. 44, W90–W97 (2016). 27141961 10.1093/nar/gkw377 PMC4987924

[72] R. C. McLeay, T. L. Bailey, Motif enrichment analysis: A unified framework and an evaluation on ChIP data. BMC Bioinformatics 11, 165 (2010). 20356413 10.1186/1471-2105-11-165 PMC2868005

[73] C. E. Grant, T. L. Bailey, W. S. Noble, FIMO: Scanning for occurrences of a given motif. Bioinformatics 27, 1017–1018 (2011). 21330290 10.1093/bioinformatics/btr064 PMC3065696

[74] Y. Perez-Riverol, A. Csordas, J. Bai, M. Bernal-Llinares, S. Hewapathirana, D. J. Kundu, A. Inuganti, J. Griss, G. Mayer, M. Eisenacher, E. Pérez, J. Uszkoreit, J. Pfeuffer, T. Sachsenberg, S. Yilmaz, S. Tiwary, J. Cox, E. Audain, M. Walzer, A. F. Jarnuczak, T. Ternent, A. Brazma, J. A. Vizcaíno, The PRIDE database and related tools and resources in 2019: Improving support for quantification data. Nucleic Acids Res. 47, D442–D450 (2019). 30395289 10.1093/nar/gky1106 PMC6323896

