# Supplementary material for: Genome-wide screening identifies Polycomb repressive complex 1.3 as an essential regulator of human naïve pluripotent cell reprogramming
Source: Sci Adv. 2022 Mar 25;8(12):eabk0013. doi: 10.1126/sciadv.abk0013 (PMC8956265; doi:10.1126/sciadv.abk0013)
Supplement: Supplementary file 1 — Figs. S1 to S9 [file sciadv.abk0013_sm.pdf]

Science Advances
AAAS

Supplementary Materials for

Genome-wide screening identifies Polycomb repressive complex 1.3 as an essential regulator of human naïve pluripotent cell reprogramming

Amanda J. Collier, Adam Bendall, Charlene Fabian, Andrew A. Malcolm, Katarzyna Tilgner, Claudia I. Semprich, Katarzyna Wojdyla, Paola Serena Nisi, Kamal Kishore, Valar Nila Roamio Franklin, Bahar Mirshekar-Syahkal, Clive D'Santos, Kathrin Plath, Kosuke Yusa, Peter J. Rugg-Gunn*

*Corresponding author. Email: peter.rugg-gunn@babraham.ac.uk

Published 25 March 2022, *Sci. Adv.* **8**, eabk0013 (2022)
DOI: 10.1126/sciadv.abk0013

The PDF file includes:

Figs. S1 to S9
Legends for data S1 to S4

Other Supplementary Material for this manuscript includes the following:

Data S1 to S4

Fig. S1

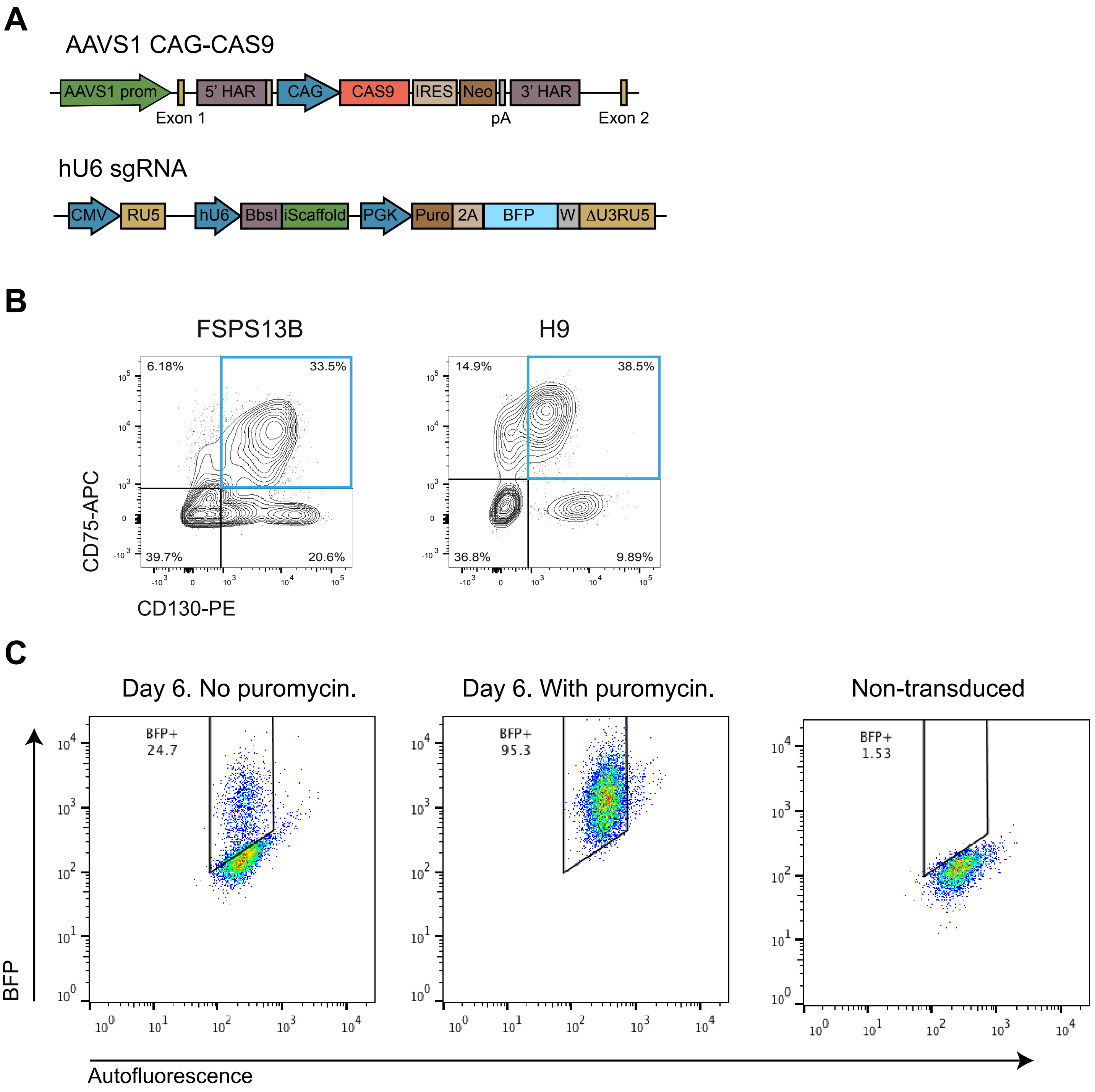
A
AAVS1 CAG-CAS9
AAVS1 prom
5' HAR
CAG
CAS9
IRES
Neo
3' HAR
Exon 1
pA
Exon 2
hU6 sgRNA
CMV
RU5
hU6
BbsI
iScaffold
PGK
Puro
2A
BFP
W
ΔU3RU5
B
FSPS13B
H9
6.18%
33.5%
39.7%
20.6%
14.9%
38.5%
36.8%
9.89%
CD75-APC
CD130-PE
C
Day 6. No puromycin.
Day 6. With puromycin.
Non-transduced
BFP+
24.7
BFP+
95.3
BFP+
1.53
BFP
Autofluorescence

Fig. S1. Additional information about the setup of the CRISPR-Cas9 screen. (**A**), Schematic diagrams show the Cas9 and gRNA expression constructs. Cas9 expression is driven by a CAG promoter and this unit is stably integrated into the *AAVS1* safe harbour locus in the FSPS13B primed PSC line. The gRNA scaffolds are driven from a hU6 promoter within a lentivirus expression construct; the plasmid also expresses puromycin and bright fluorescent protein (BFP) from a PGK promoter. (**B**), Flow cytometry contour plots confirm that the Cas9-expressing primed PSC can reprogramme into nascent naïve cells with the expected efficiency (compared to unmodified primed PSC lines). (**C**), Flow cytometry dot plots show that the majority (>95%) of transduced and puromycin-selected primed PSCs express the BFP-containing gRNA plasmid. Puromycin was applied for three days (days 3-6) following lentiviral transduction. Parallel cultures without puromycin selection, and non-transduced cells, are shown for comparison.

Fig. S2

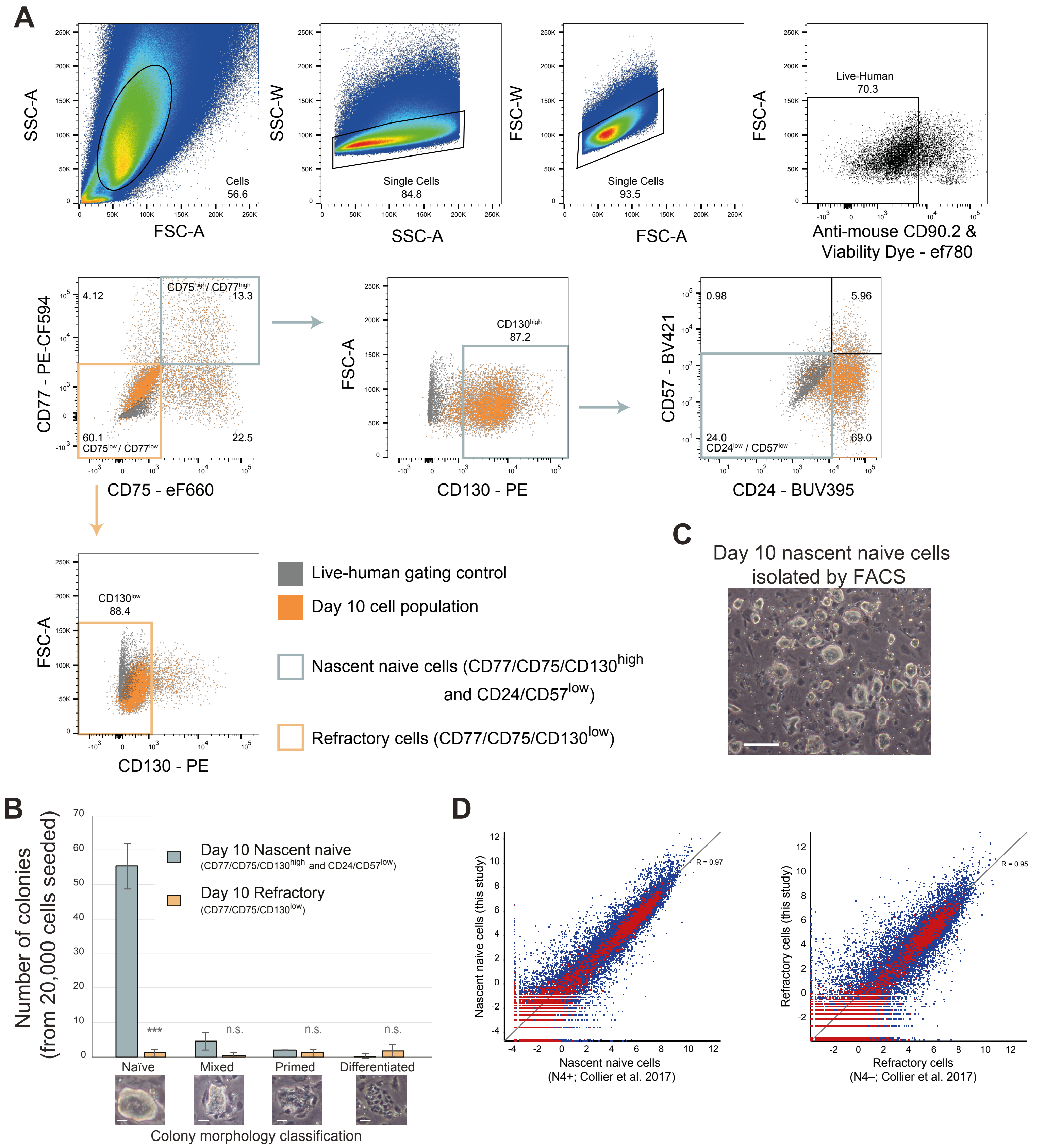
A
SSC-A
FSC-A
Cells
56.6
SSC-W
SSC-A
Single Cells
84.8
FSC-W
FSC-A
Single Cells
93.5
FSC-A
Live-Human
70.3
Anti-mouse CD90.2 &
Viability Dye - ef780
CD77 - PE-CF594
CD75 - eF660
4.12
CD75high / CD77high
13.3
60.1
CD75low / CD77low
22.5
FSC-A
CD130 - PE
CD130high
87.2
CD57 - BV421
CD24 - BUV395
0.98
5.96
24.0
CD24low / CD57low
69.0
CD130low
88.4
Live-human gating control
Day 10 cell population
Nascent naive cells (CD77/CD75/CD130high
and CD24/CD57low)
Refractory cells (CD77/CD75/CD130low)
C
Day 10 nascent naive cells
isolated by FACS
B
Number of colonies
(from 20,000 cells seeded)
Day 10 Nascent naive
(CD77/CD75/CD130high and CD24/CD57low)
Day 10 Refractory
(CD77/CD75/CD130low)
***
n.s.
n.s.
n.s.
Naïve
Mixed
Primed
Differentiated
Colony morphology classification
D
Nascent naive cells (this study)
Nascent naive cells
(N4+; Collier et al. 2017)
R = 0.97
Refractory cells (this study)
Refractory cells
(N4–; Collier et al. 2017)
R = 0.95

Fig. S2: Additional information about the cell-sorted populations. (**A**), Flow cytometry gating strategy used to isolate the nascent naïve and refractory cell populations at day 10 of 5iLA-mediated reprogramming. The first three gates identify single cells. The fourth gate identifies live, human cells; mouse 'feeder' embryonic fibroblasts are positive for Cd90.2 and are removed at this step, along with dead cells that are positive for the viability dye. The remaining gates apply a stringent strategy to identify nascent naïve PSCs (CD75/CD77/CD130–high & CD24/CD57–low; ~3% of live, human cells in the population) and the refractory cells (CD75/CD77/CD130–low; ~53% of the live, human cells in the population). (**B**), Chart shows that nascent naïve cells isolated using the above strategy give rise to naïve PSC colonies when re-plated in culture, whereas the refractory cells are unable to form naïve PSC colonies. Data show mean from three experiments; error bars, s.d.; ***, $p<0.0001$; n.s., not significant; Student's t-test comparing nascent naïve with refractory populations. (**C**), Phase contrast image of the cultures following the re-plating of isolated nascent naïve PSCs. Scale bar, 100μm. (**D**), Scatter plots show the high correlations ($R=0.95$) in transcript expression between the cell-sorted nascent naïve PSCs (left) and refractory cells (right) in this current study with a prior study (Collier et al., 2017).

Fig. S3

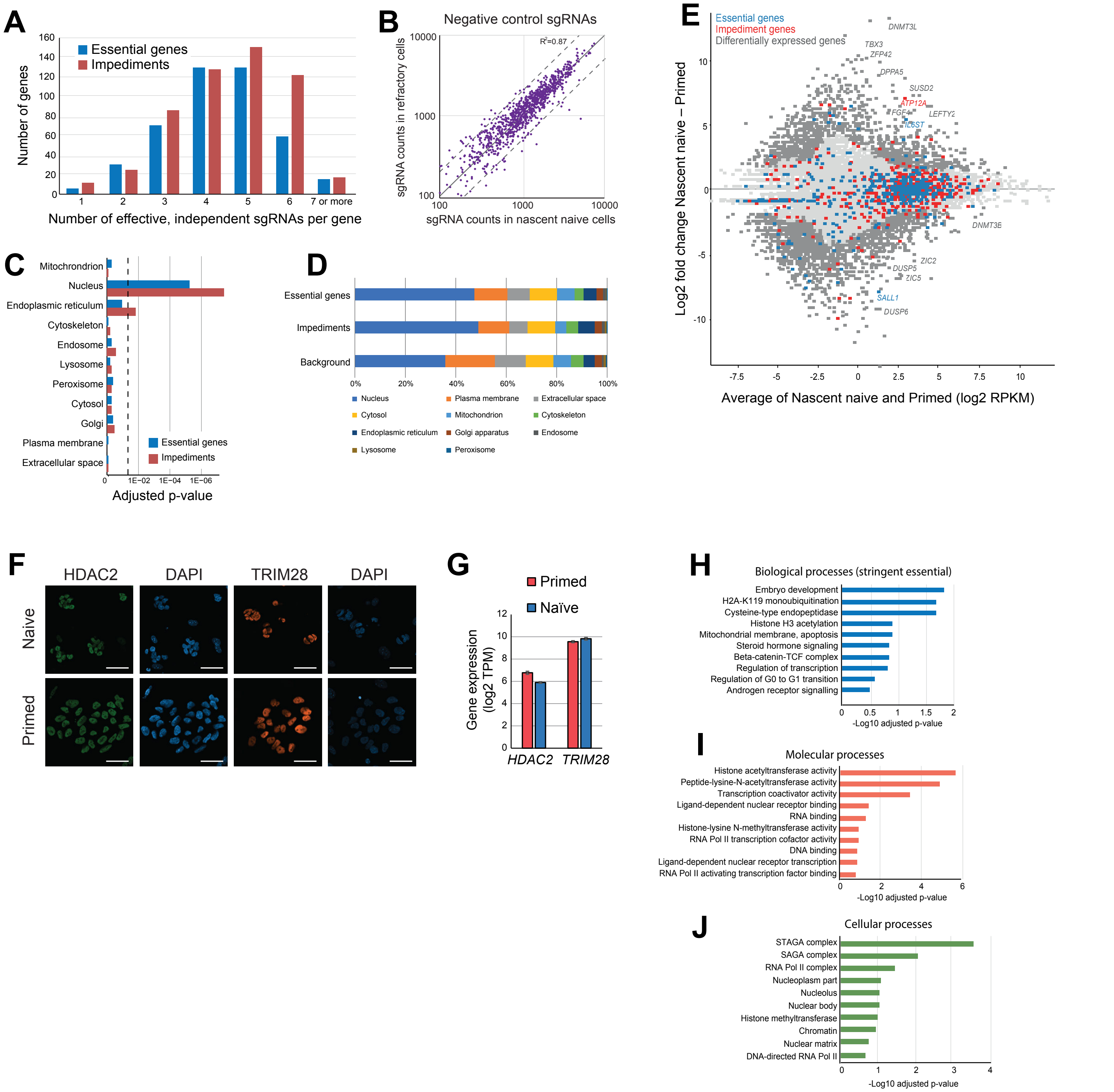
A
Number of genes
Essential genes
Impediments
1
2
3
4
5
6
7 or more
Number of effective, independent sgRNAs per gene
B
Negative control sgRNAs
R^2=0.87
sgRNA counts in refractory cells
sgRNA counts in nascent naive cells
E
Essential genes
Impediment genes
Differentially expressed genes
DNMT3L
TBX3
ZFP42
DPPA5
SUSD2
ATP12A
FGF4
LEFTY2
IL6ST
DNMT3B
ZIC2
DUSP5
ZIC5
SALL1
DUSP6
Log2 fold change Nascent naive – Primed
Average of Nascent naive and Primed (log2 RPKM)
C
Mitochrondrion
Nucleus
Endoplasmic reticulum
Cytoskeleton
Endosome
Lysosome
Peroxisome
Cytosol
Golgi
Plasma membrane
Extracellular space
Essential genes
Impediments
Adjusted p-value
D
Essential genes
Impediments
Background
Nucleus
Plasma membrane
Extracellular space
Cytosol
Mitochondrion
Cytoskeleton
Endoplasmic reticulum
Golgi apparatus
Endosome
Lysosome
Peroxisome
F
HDAC2
DAPI
TRIM28
DAPI
Naive
Primed
G
Primed
Naïve
Gene expression (log2 TPM)
HDAC2
TRIM28
H
Biological processes (stringent essential)
Embryo development
H2A-K119 monoubiquitination
Cysteine-type endopeptidase
Histone H3 acetylation
Mitochondrial membrane, apoptosis
Steroid hormone signaling
Beta-catenin-TCF complex
Regulation of transcription
Regulation of G0 to G1 transition
Androgen receptor signalling
-Log10 adjusted p-value
I
Molecular processes
Histone acetyltransferase activity
Peptide-lysine-N-acetyltransferase activity
Transcription coactivator activity
Ligand-dependent nuclear receptor binding
RNA binding
Histone-lysine N-methyltransferase activity
RNA Pol II transcription cofactor activity
DNA binding
Ligand-dependent nuclear receptor transcription
RNA Pol II activating transcription factor binding
-Log10 adjusted p-value
J
Cellular processes
STAGA complex
SAGA complex
RNA Pol II complex
Nucleoplasm part
Nucleolus
Nuclear body
Histone methyltransferase
Chromatin
Nuclear matrix
DNA-directed RNA Pol II
-Log10 adjusted p-value

Fig. S3. Further analysis of essential and impediment genes. (**A**) Chart shows that >92% of all essential and impediment genes were associated with three or more effective, independent gRNAs. (**B**) Scatter plot shows that the gRNAs targeting negative control regions were distributed equally in both cell populations (>95% of control gRNAs had a fold change <2; n=1004). (**C**) Chart shows that the essential and impediment genes encode proteins that are strongly enriched for factors localised in the nucleus, compared to a background gene set comprising all genes in the screen. Adjusted p-values are calculated using a Fisher exact test. (**D**) Chart shows the distribution across different cellular compartments for essential, impediment and background factors. (**E**) MA plot shows that the majority (>97%) of essential and impediment genes are expressed at similar levels when comparing nascent naïve PSCs at day 10 of reprogramming and the starting primed PSC population at day 0. Transcriptional data are shown for each gene (represented by a dot), comparing nascent naive and primed PSC populations; dark grey, differentially expressed; light grey, all other genes. Essential and impediment genes are coloured in blue and red, respectively. (**F**) Immunofluorescence microscopy shows unchanged expression and localisation of HDAC2 (impediment factor) and TRIM28 (essential factor) when comparing between naive and primed PSCs. Scale bars, 50 µm. (**G**) Gene expression analysis shows similar transcript levels for *HDAC2* and for *TRIM28* in naive and primed PSCs. Mean with S.D.; n=3 biological replicates. Data are from Collier at al., 2017. (**H-J**) Charts show the adjusted p-values for essential genes in the indicated gene ontology categories. Panel **(H)** is subsetted to the 382 essential genes that are not required for primed PSC proliferation (identified in Fig. 2 and Data S2). Adjusted p-values are calculated using a Fisher exact test.

Fig. S4

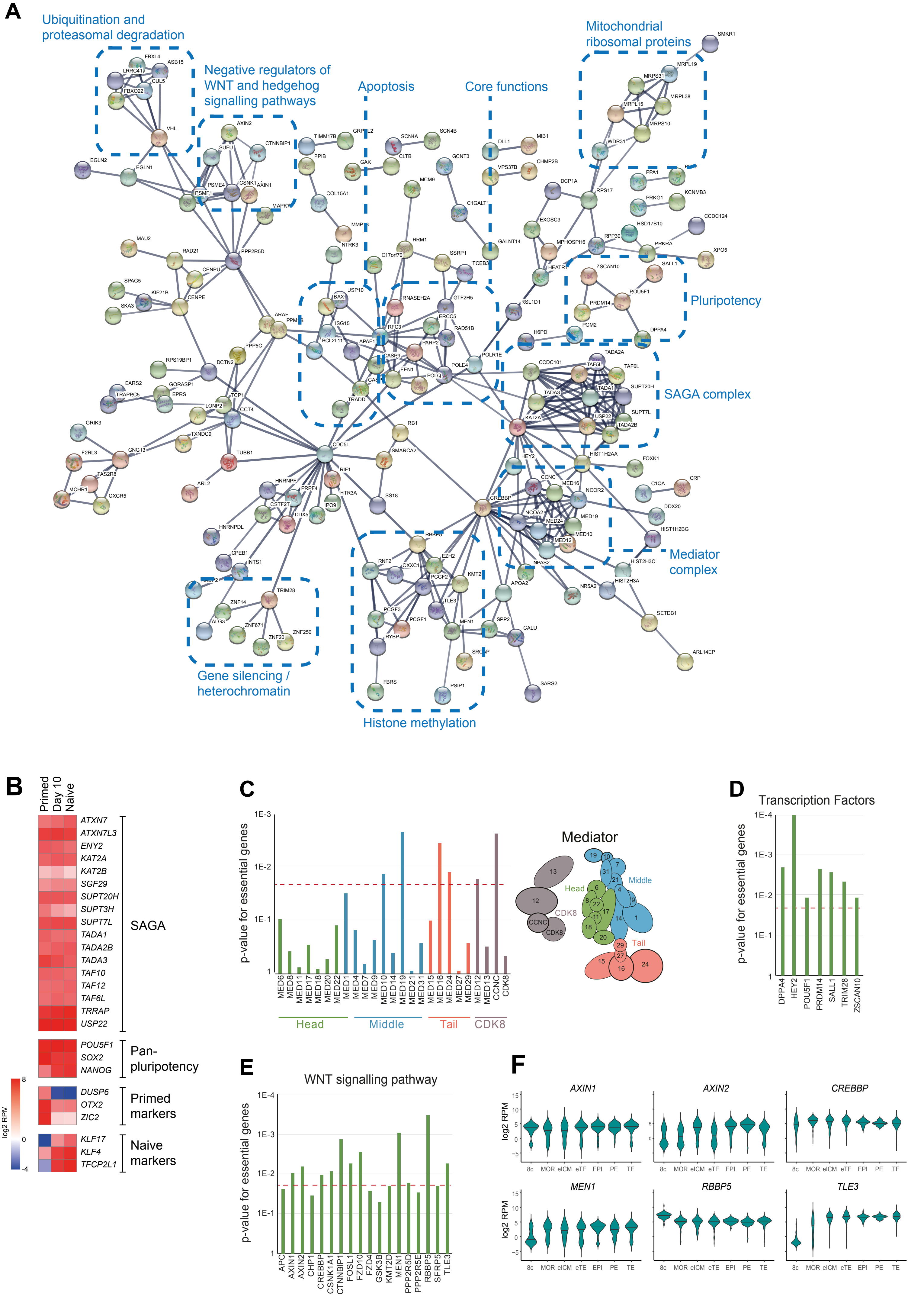

A
Ubiquitination and proteasomal degradation
Negative regulators of WNT and hedgehog signalling pathways
Apoptosis
Core functions
Mitochondrial ribosomal proteins
Pluripotency
SAGA complex
Mediator complex
Gene silencing / heterochromatin
Histone methylation
B
Primed
Day 10
Naive
ATXN7
ATXN7L3
ENY2
KAT2A
KAT2B
SGF29
SUPT20H
SUPT3H
SUPT7L
TADA1
TADA2B
TADA3
TAF10
TAF12
TAF6L
TRRAP
USP22
SAGA
POU5F1
SOX2
NANOG
Pan-pluripotency
DUSP6
OTX2
ZIC2
Primed markers
KLF17
KLF4
TFCP2L1
Naive markers
log2 RPM
C
p-value for essential genes
Mediator
Head
Middle
Tail
CDK8
D
Transcription Factors
p-value for essential genes
DPPA4
HEY2
POU5F1
PRDM14
SALL1
TRIM28
ZSCAN10
E
WNT signalling pathway
p-value for essential genes
APC
AXIN1
AXIN2
CHP1
CREBBP
CSNK1A1
CTNNBIP1
FOSL1
FZD10
FZD4
GSK3B
KMT2D
MEN1
PPP2R5D
PPP2R5E
RBBP5
SFRP5
TLE3
F
AXIN1
AXIN2
CREBBP
MEN1
RBBP5
TLE3
log2 RPM
8c
MOR
eICM
eTE
EPI
PE
TE

Fig. S4. Protein-protein interaction networks for all essential factors and example complexes and pathways.
(**A**) STRING-based visualisation of protein-protein interactions for factors encoded by essential genes. The confidence cut-off for showing interaction links has been set to 'high' (0.700), text mining switched off, and disconnected nodes are hidden. The network has 380 edges, which is significantly more than expected (p=1e-09). (**B**) Heatmap shows transcript expression levels at day 0 (primed PSCs), day 10 (nascent naïve PSCs) and day 34 (established naïve PSC lines). Data are from Collier et al., 2017. (**C-E**) Charts show the p-values of (**C**) Mediator complex members, (**D**) selected transcription factors identified in this study as being essential for naive PSC, and (**E**) WNT signalling pathway components, as a measure of their depletion in the nascent naive cell population following the CRISPR-Cas9 screen. The red line indicates p=0.02 as a significance cut-off (permutation test). Schematic of the Mediator complex is also shown; bold lines indicate components classified as an essential gene. (**F**) Plots show the distribution in expression levels of the indicated WNT pathway genes in single cells throughout human preimplantation embryo development. 8c, 8-cell stage; MOR, morula; eICM, early inner cell mass; eTE, early trophectoderm; EPI, epiblast; PE, primitive endoderm; TE, trophectoderm. Data are from Petropoulos et al., 2016 with annotation from Stirparo et al., 2018.

Fig. S5

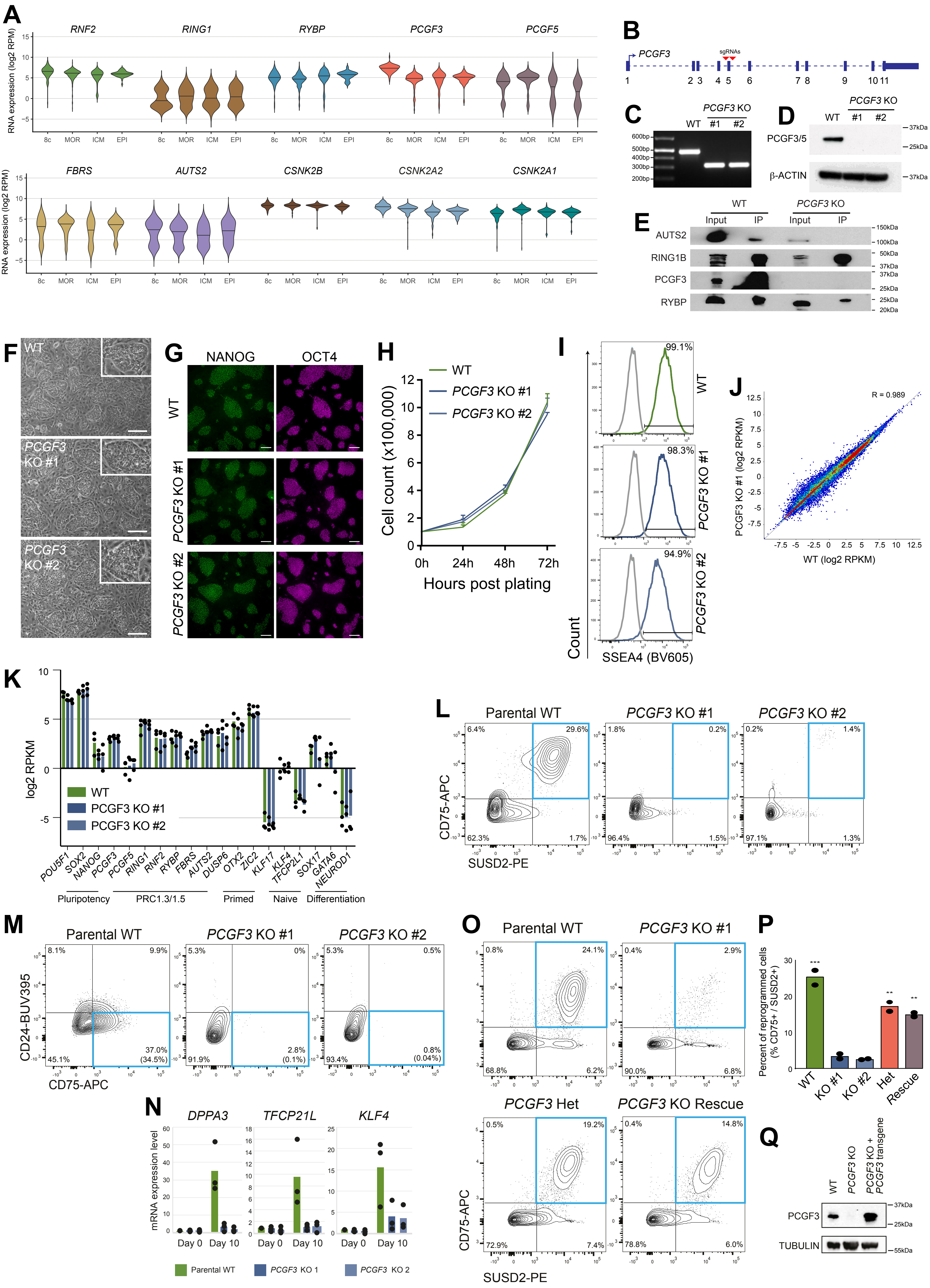
A
RNF2
RING1
RYBP
PCGF3
PCGF5
RNA expression (log2 RPM)
8c MOR ICM EPI
FBRS
AUTS2
CSNK2B
CSNK2A2
CSNK2A1
B
PCGF3
sgRNAs
1 2 3 4 5 6 7 8 9 10 11
C
PCGF3 KO
WT #1 #2
600bp 500bp 400bp 300bp 200bp
D
PCGF3 KO
WT #1 #2
PCGF3/5
β-ACTIN
37kDa 25kDa 37kDa
E
WT PCGF3 KO
Input IP Input IP
AUTS2
RING1B
PCGF3
RYBP
150kDa 100kDa 50kDa 37kDa 37kDa 25kDa 25kDa 20kDa
F
WT
PCGF3 KO #1
PCGF3 KO #2
G
NANOG OCT4
WT
PCGF3 KO #1
PCGF3 KO #2
H
WT
PCGF3 KO #1
PCGF3 KO #2
Cell count (x100,000)
0h 24h 48h 72h
Hours post plating
I
99.1%
98.3%
94.9%
WT
PCGF3 KO #1
PCGF3 KO #2
Count
SSEA4 (BV605)
J
R = 0.989
PCGF3 KO #1 (log2 RPKM)
WT (log2 RPKM)
K
log2 RPKM
WT
PCGF3 KO #1
PCGF3 KO #2
POU5F1 SOX2 NANOG PCGF3 PCGF5 RING1 RNF2 RYBP FBRS AUTS2 DUSP6 OTX2 ZIC2 KLF17 KLF4 TFCP2L1 SOX17 GATA6 NEUROD1
Pluripotency PRC1.3/1.5 Primed Naive Differentiation
L
Parental WT
PCGF3 KO #1
PCGF3 KO #2
6.4% 29.6% 62.3% 1.7%
1.8% 0.2% 96.4% 1.5%
0.2% 1.4% 97.1% 1.3%
CD75-APC
SUSD2-PE
M
Parental WT
PCGF3 KO #1
PCGF3 KO #2
8.1% 9.9% 45.1% 37.0% (34.5%)
5.3% 0% 91.9% 2.8% (0.1%)
5.3% 0.5% 93.4% 0.8% (0.04%)
CD24-BUV395
CD75-APC
N
DPPA3
TFCP21L
KLF4
mRNA expression level
Day 0 Day 10
Parental WT
PCGF3 KO 1
PCGF3 KO 2
O
Parental WT
PCGF3 KO #1
0.8% 24.1% 68.8% 6.2%
0.4% 2.9% 90.0% 6.8%
PCGF3 Het
PCGF3 KO Rescue
0.5% 19.2% 72.9% 7.4%
0.4% 14.8% 78.8% 6.0%
CD75-APC
SUSD2-PE
P
Percent of reprogrammed cells (% CD75+ / SUSD2+)
***
**
**
WT KO #1 KO #2 Het Rescue
Q
WT
PCGF3 KO
PCGF3 KO + PCGF3 transgene
PCGF3
TUBULIN
37kDa 25kDa 55kDa

Fig. S5. Further analysis of PRC1.3 expression and function. (**A**), Plots show the distribution in expression levels of the indicated PRC1.3 components in single cells throughout human preimplantation embryo development. 8c, 8-cell stage; MOR, morula; eICM, early inner cell mass; EPI, epiblast. Data are from Petropoulos et al., 2016 with annotation from Stirparo et al., 2018. (**B**), Schematic of *PCGF3* exon structure. The red triangles indicate the position of the gRNAs used. (**C**), Electrophoresis image of PCR-based genotyping using primers that flank the deleted region within *PCGF3*. Wild-type (WT) parental primed PSCs show a band at the expected size. The smaller band in the two knockout (KO) primed PSC lines indicate homozygous deletion of the targeted exon. The size of the DNA fragments in the ladder are shown in bp. (**D**), Western blot shows the absence of detectable PCGF3 protein in two KO primed PSC lines. β–ACTIN, loading control. (**E**), Co-immunoprecipitation with RING1B antibody shows that the PRC1.3 complex dissociates in *PCGF3* KO primed PSC. (**F**), Phase contrast images show the similar cell morphology of WT and *PCGF3* KO #1 and #2 primed PSCs. The WT cells are untargeted parental H9 cells. Scale bars, 250 μm. (**G**), Immunofluorescence microscopy images show the uniform expression of OCT4 and NANOG in WT and *PCGF3* KO primed PSC lines. Scale bars, 250 μm. (**H**), Chart shows the similar proliferation rates when comparing WT with *PCGF3* KO primed PSCs. (**I**), Flow cytometry histograms of SSEA4 expression in WT and *PCGF3* KO primed PSC lines. Grey lines show samples omitting the SSEA4 antibody. (**J**), Scatter plot shows the high correlation (R=0.989) in transcript expression between WT and *PCGF3* KO clone #1. Similar results were obtained for the *PCGF3* KO clone #2 (R=0.988; not shown). (**K**), Chart shows the similarity in transcript levels of marker genes when comparing between WT and *PCGF3* KO primed PSCs. (**L**), Flow cytometry contour plots of parental WT and *PCGF3* KO PSCs at day 14 of naive PSC reprogramming (5iLA conditions) labelled with antibodies against two naive PSC markers. Successfully reprogrammed naive cells appear in the upper right quadrant. Data are representative of three independent experiments. (**M**), Flow cytometry contour plots of parental WT and *PCGF3* KO PSCs at day 24 of naive PSC reprogramming (5i/L/A conditions). Successfully reprogrammed naive cells appear in the lower right quadrant. The numbers in each quadrant show the percentage of cells out of the live human cells in the sample; the numbers in parentheses show the percentage of cells out of all single cells in the sample (therefore including dead and dying cells, and mouse feeder cells). Data are representative of two independent experiments. (**N**), RT-qPCR data show the induction of naive PSC marker genes in WT, but not *PCGF3* KO cells, at 10 day of reprogramming. Individual data points are shown for three independent experiments. (**O**), Flow cytometry contour plots of parental WT, *PCGF3* heterozygous (Het), *PCGF3* KO and *PCGF3* KO expressing a *PCGF3* transgene (Rescue) PSCs at day 10 of naive PSC reprogramming (CR conditions) labelled with antibodies against two naive PSC markers. Successfully reprogrammed naive cells appear in the upper right quadrant. Data are representative of two KO lines and two independent experiments. (**P**), Summary of flow cytometry data shown in Fig. S5O. Samples are compared to each of the *PCGF3* KO cell lines using a one-way ANOVA with Tukey correction ($F_{4,5} = 60.19$). **, p<0.01; ***, p=0.0005. Two independent experiments.(**Q**), Western blot shows the expression of PCGF3 protein in the 'rescue' primed PSC line. TUBULIN, loading control.

Fig. S6

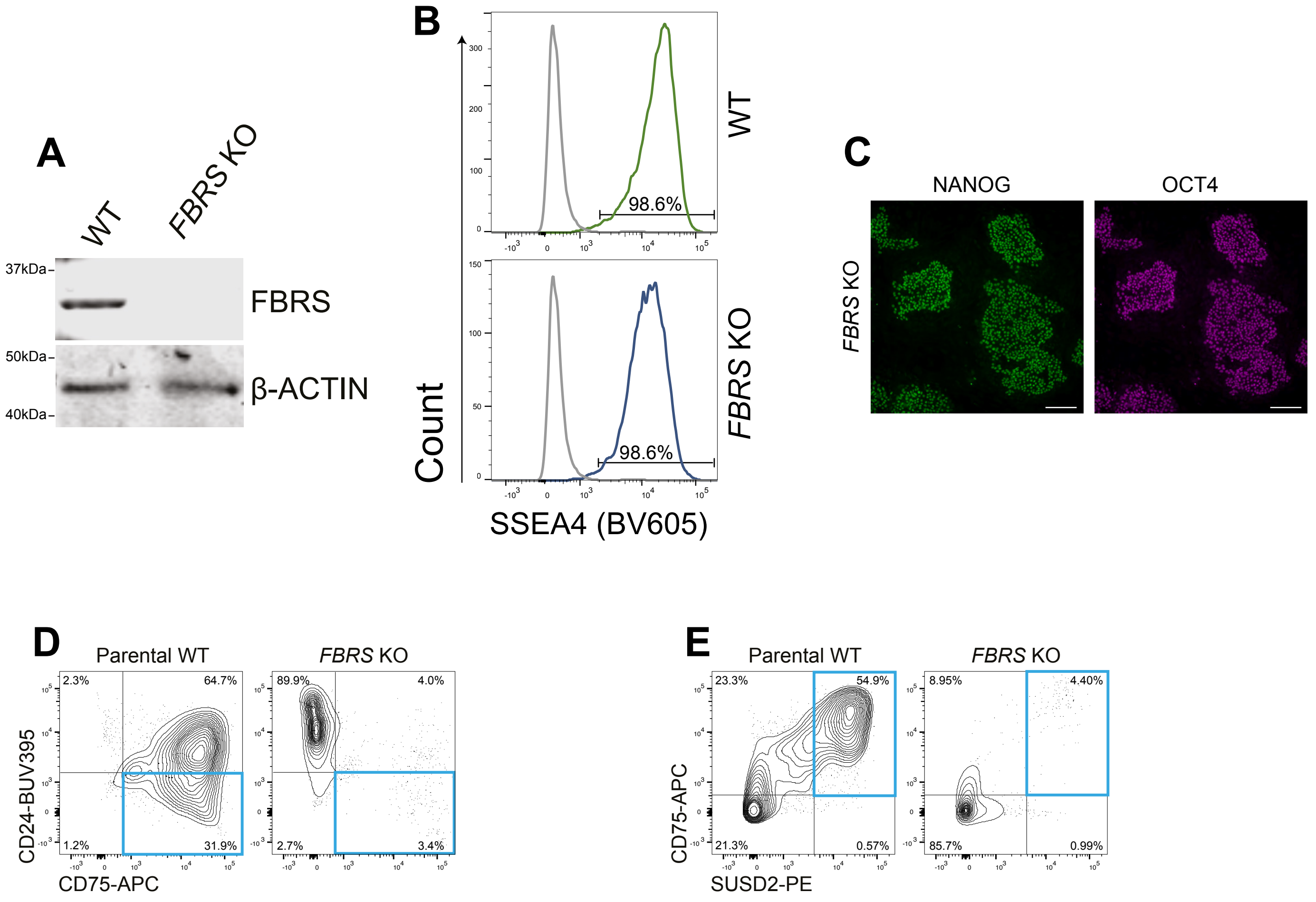
A
WT
FBRS KO
37kDa
50kDa
40kDa
FBRS
β-ACTIN
B
WT
FBRS KO
Count
98.6%
98.6%
SSEA4 (BV605)
C
NANOG
OCT4
FBRS KO
D
Parental WT
FBRS KO
2.3%
64.7%
1.2%
31.9%
89.9%
4.0%
2.7%
3.4%
CD24-BUV395
CD75-APC
E
Parental WT
FBRS KO
23.3%
54.9%
21.3%
0.57%
8.95%
4.40%
85.7%
0.99%
CD75-APC
SUSD2-PE

Fig. S6. Characterisation of *FBRS* KO PSCs and reprogramming defect. (**A**), Western blot shows the absence of detectable FBRS protein in the KO primed PSC line. β−ACTIN, loading control. (**B**), Flow cytometry histograms of SSEA4 expression in WT and *FBRS* KO primed PSC lines. Grey lines indicate samples omitting the SSEA4 antibody. (**C**), Immunofluorescence microscopy images show the uniform expression of OCT4 and NANOG in WT and *FBRS* KO primed PSC lines. Scale bars, 250 μm. (**D**), Flow cytometry contour plots of parental WT and *FBRS* KO PSCs at day 12 of naive PSC reprogramming (CR conditions). Successfully reprogrammed naive cells appear in the lower right quadrant. Data are representative of two independent experiments. (**E**), Flow cytometry contour plots of WT and *FBRS* KO cells at day 12 of naive PSC reprogramming (CR conditions) labelled with two naive-specific antibodies. Successfully reprogrammed naive cells appear in the upper right quadrant. Data are representative of two independent experiments.

Fig. S7

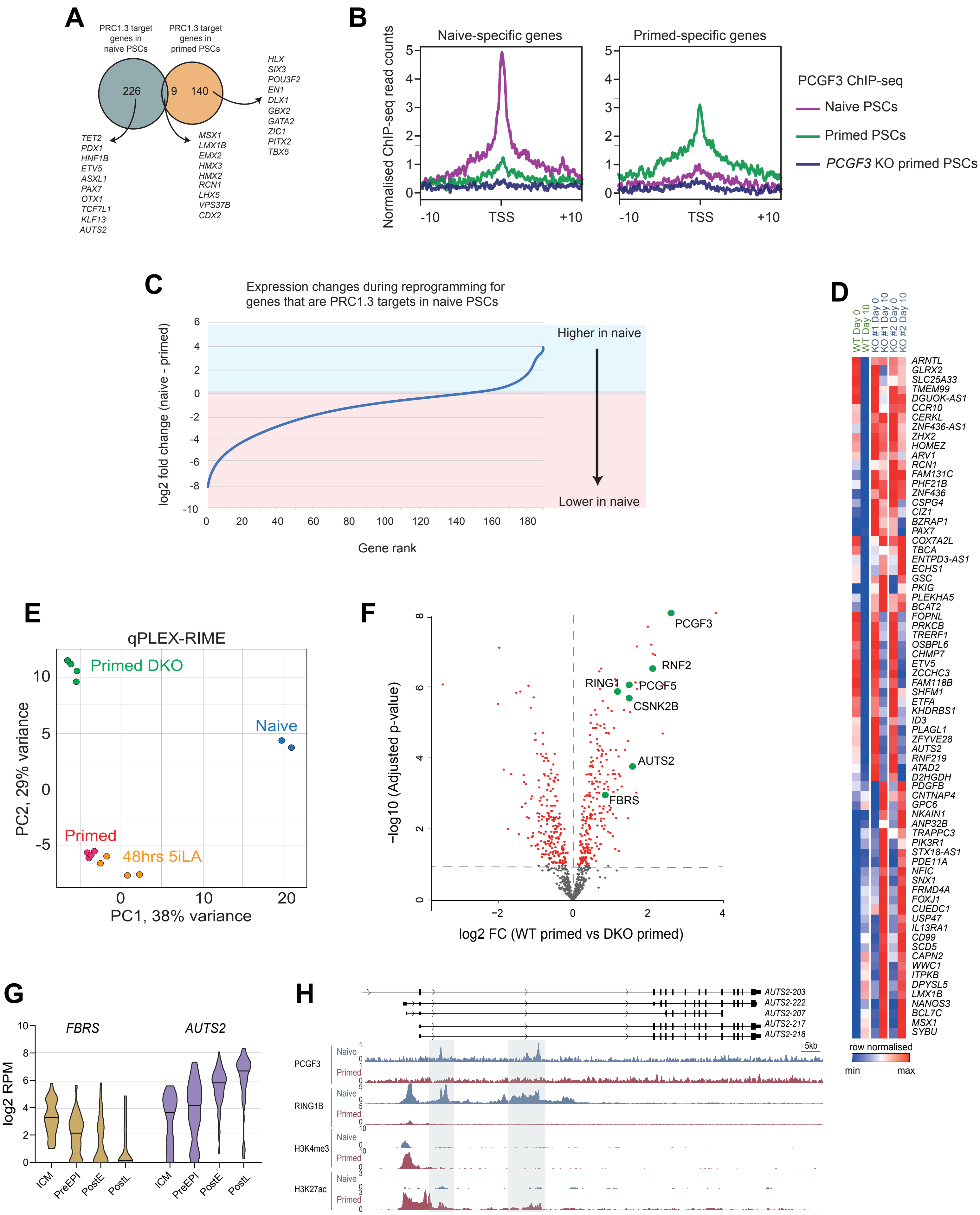

A
PRC1.3 target genes in naive PSCs
PRC1.3 target genes in primed PSCs
226
9
140
HLX
SIX3
POU3F2
EN1
DLX1
GBX2
GATA2
ZIC1
PITX2
TBX5
TET2
PDX1
HNF1B
ETV5
ASXL1
PAX7
OTX1
TCF7L1
KLF13
AUTS2
MSX1
LMX1B
EMX2
HMX3
HMX2
RCN1
LHX5
VPS37B
CDX2
B
Naive-specific genes
Primed-specific genes
Normalised ChIP-seq read counts
-10
TSS
+10
PCGF3 ChIP-seq
Naive PSCs
Primed PSCs
PCGF3 KO primed PSCs
C
Expression changes during reprogramming for genes that are PRC1.3 targets in naive PSCs
log2 fold change (naive - primed)
Higher in naive
Lower in naive
Gene rank
D
WT Day 0
WT Day 10
KO #1 Day 0
KO #1 Day 10
KO #2 Day 0
KO #2 Day 10
ARNTL
GLRX2
SLC25A33
TMEM99
DGUOK-AS1
CCR10
CERKL
ZNF436-AS1
ZHX2
HOMEZ
ARV1
RCN1
FAM131C
PHF21B
ZNF436
CSPG4
CIZ1
BZRAP1
PAX7
COX7A2L
TBCA
ENTPD3-AS1
ECHS1
GSC
PKIG
PLEKHA5
BCAT2
FOPNL
PRKCB
TRERF1
OSBPL6
CHMP7
ETV5
ZCCHC3
FAM118B
SHFM1
ETFA
KHDRBS1
ID3
PLAGL1
ZFYVE28
AUTS2
RNF219
ATAD2
D2HGDH
PDGFB
CNTNAP4
GPC6
NKAIN1
ANP32B
TRAPPC3
PIK3R1
STX18-AS1
PDE11A
NFIC
SNX1
FRMD4A
FOXJ1
CUEDC1
USP47
IL13RA1
CD99
SCD5
CAPN2
WWC1
ITPKB
DPYSL5
LMX1B
NANOS3
BCL7C
MSX1
SYBU
row normalised
min
max
E
qPLEX-RIME
Primed DKO
Naive
Primed
48hrs 5iLA
PC2, 29% variance
PC1, 38% variance
F
PCGF3
RNF2
RING1
PCGF5
CSNK2B
AUTS2
FBRS
−log10 (Adjusted p-value)
log2 FC (WT primed vs DKO primed)
G
FBRS
AUTS2
log2 RPM
ICM
PreEPI
PostE
PostL
H
AUTS2-203
AUTS2-222
AUTS2-207
AUTS2-217
AUTS2-218
5kb
PCGF3
RING1B
H3K4me3
H3K27ac
Naive
Primed

Fig. S7. Further information related to the function and regulation of PRC1.3. (**A**), Venn diagrams show the overlap in PRC1.3 target genes in naïve and primed PSCs. (**B**), Metaplot of PCGF3 ChIP-seq signal over naive-specific and primed-specific target genes (20kb window centered on the transcriptional start sites) in naive PSCs, primed PSCs and *PCGF3* KO primed PSCs. (**C**), Chart shows the change in expression levels of PRC1.3 target genes between naïve and primed PSCs. Genes are ranked according to their fold change. (**D**), Heatmap shows the row normalised transcript levels for a subset of PRC1.3 target genes in WT and PCGF3 KO cell populations at days 0 and 10 of naïve reprogramming (5i/L/A conditions). The majority of the genes are transcriptionally repressed in day 10 WT cells, but are aberrantly expressed in day 10 KO cells in two separate clonal lines. (**E**), PCA of the qPLEX-RIME data. Each circle represents an independent biological replicate. DKO, *PCGF3/PCGF5* double KO cells; 48hrs 5i/L/A, primed PSCs cultured in 5i/L/A reprogramming conditions for 48 hours. (**F**), Volcano plot of qPLEX-RIME data shows the FC in abundance and the associated p-values of PCGF3-interacting proteins when comparing WT and DKO primed PSCs. Proteins with an adjusted p-value < 0.1, red dots; proteins with an adjusted p-value > 0.1, grey dots; PRC1.3 core components, green circles. (**G**), Plots show the distribution in expression levels of *FBRS* and *AUTS2* in single cells during cynomolgus monkey embryo development. ICM, inner cell mass; PreEPI, preimplantation epiblast; PostE, early postimplantation epiblast; PostL, late postimplantation epiblast. Data are from Nakamura et al., 2016. (**H**), Genome browser representation of ChIP-seq data at the *AUTS2* locus. The two regions highlighted in blue reveal PRC1.3 (PCGF3 & RING1B) occupancy at this locus in naïve but not primed PSCs. Note also the higher levels of H3K4me3 and H3K27ac in primed PSCs, which is consistent with *AUTS2* expression in primed but not in naïve PSCs. Tracks show read counts normalised to total library size.

Fig. S8

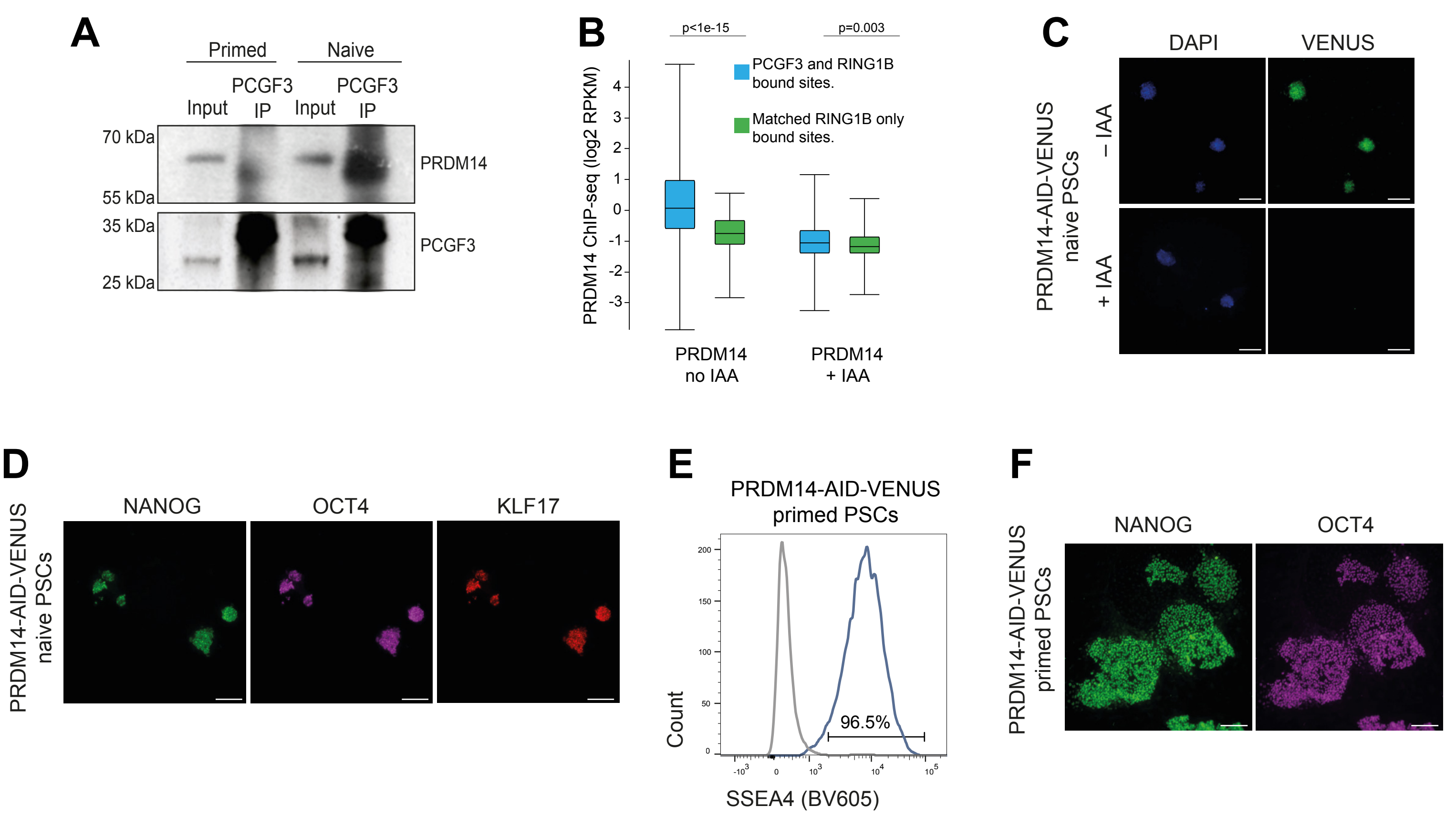
A
Primed
Naive
Input
PCGF3 IP
Input
PCGF3 IP
70 kDa
55 kDa
35 kDa
25 kDa
PRDM14
PCGF3
B
p<1e-15
p=0.003
PCGF3 and RING1B bound sites.
Matched RING1B only bound sites.
PRDM14 ChIP-seq (log2 RPKM)
PRDM14 no IAA
PRDM14 + IAA
C
DAPI
VENUS
PRDM14-AID-VENUS naive PSCs
– IAA
+ IAA
D
PRDM14-AID-VENUS naive PSCs
NANOG
OCT4
KLF17
E
PRDM14-AID-VENUS primed PSCs
96.5%
Count
SSEA4 (BV605)
F
PRDM14-AID-VENUS primed PSCs
NANOG
OCT4

Fig. S8. Cooperativity of PRDM14 and PRC1.3 in repressing target genes. (**A**), Co-immunoprecipitation and Western blot experiments confirm an interaction between PRDM14 and PCGF3 in naïve PSCs. (**B**), Box and whisker plot shows the distribution of PRDM14-VENUS-AID ChIP-seq normalised read counts in normal growth conditions (left) and following IAA-induced PRDM14 degradation (right). Blue boxes, PRC1.3-target sites (PCGF3 & RING1B; n=471); green boxes, RING1B-only sites with a matched distribution of RING1B ChIP-seq levels (n=397). The box bounds the interquartile range divided by the median (horizontal line), and Spear-style whiskers extend to the minimum and maximum of the data values. Data were compared using a two-sided Mann-Whitney test. Data are from Sybirna et al., 2019. (**C**), Immunofluorescent microscopy images in naive PSCs showing the absence of PRDM14-AID-VENUS signal following IAA treatment. Scale bars, 200 μm. (**D**), Immunofluorescence microscopy images show the uniform expression of OCT4, NANOG and KLF17 in PRDM14-AID-VENUS naive PSCs. Scale bars, 200 μm. (**E**), Flow cytometry histogram of SSEA4 expression in PRDM14-AID-VENUS primed PSCs. Grey lines show samples omitting the SSEA4 antibody. (**F**), Immunofluorescence microscopy images show the uniform expression of OCT4 and NANOG in PRDM14-AID-VENUS primed PSCs. Scale bars, 200 μm.

Fig. S9

A

H9 WT (46XX; 19/20)

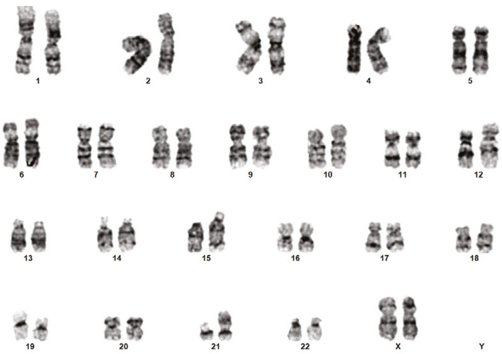

H9 PRDM14-AID-Venus (46 XX; 20/20)

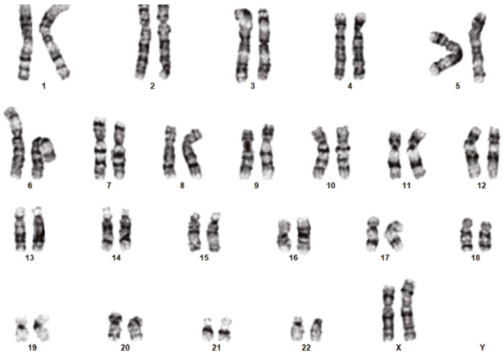

H9 PCGF3 KO #1 (46XX; 20/20)

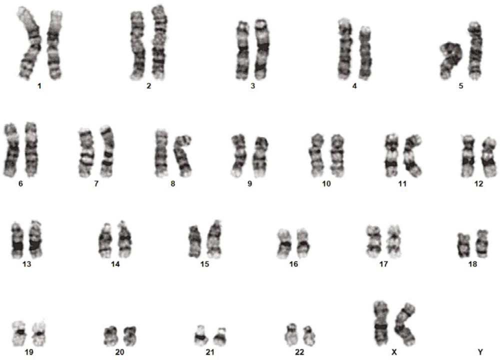

H9 PCGF3 KO #2 (46XX; 20/20)

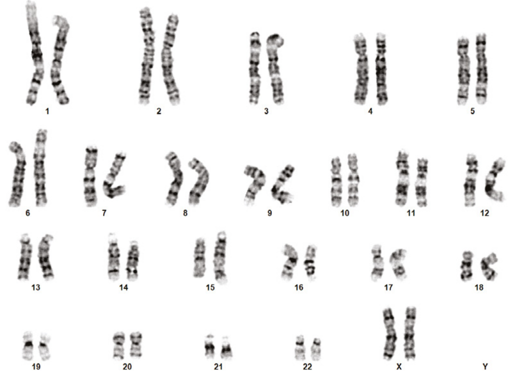

H9 PCGF3 KO Rescue (46XX; 20/20)

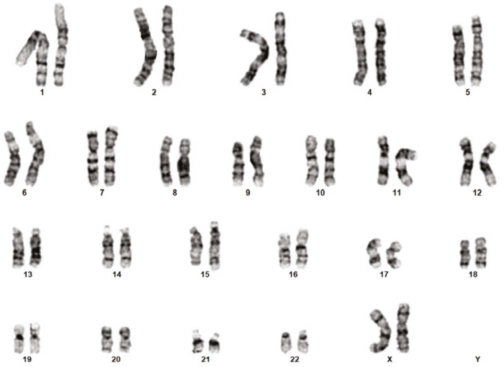

H9 FBRS KO (46XX;20/20)

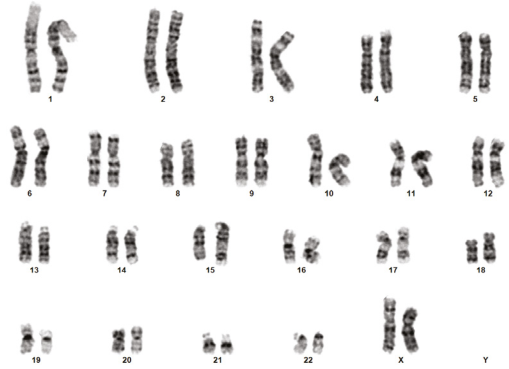

B

FSPS13B CAGCAS9 Primed

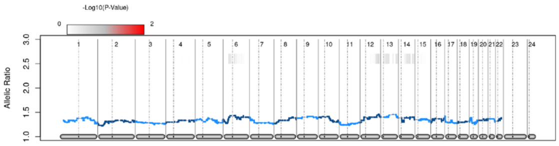

FSPS13B CAGCAS9 Day 10 refractory

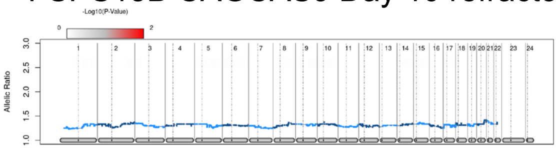

FSPS13B CAGCAS9 Day 10 nascent naive

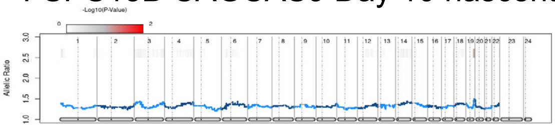

Fig. S9. Karyotype information for cell lines used in this study. (**A**), Representative spreads of G-banded chromosomes for the indicated cell lines. Twenty cells were examined for each cell line. (**B**), eSNP-Karyotyping analysis of the FSPS13B CAGCAS9 primed, refractory and nascent naive cells based on their RNA-seq profiles from the CRISPR screen. Panels show the moving average plot of SNP allelic ratios across all chromosomes. Note that the X and Y chromosomes, labelled as chromosomes "23" and "24", are excluded from the analysis. Red/grey tracks above the moving average lines indicate the FDR-corrected p-values following a one-tailed t-test comparing the SNP major/minor values in each window to the values of the entire genome.

Data S1. gRNA counts in nascent naive and refractory samples. Sequencing-based counts of each gRNA (n=113,525) in the nascent naive and refractory samples, and fold-change in gRNAs and genes between the two cell populations.

Data S2. Gene-level summary of CRISPR Cas9 screen results. Genes ranked by *P*-value derived from the robust rank aggregation method, as implemented in MAGeCK (*33*). Negative selection refers to genes depleted in the nascent naive cell population (identifying essential genes) and positive selection refers to genes enriched in the nascent naive cell population (identifying impediment genes). The FDR is calculated from the empirical permutation *P*-values using the Benjamini-Hochberg procedure. The other sheets list all essential reprogramming genes (n=446), essential reprogramming genes with the genes that are required for primed PSC proliferation removed (n=382), and reprogramming impediment genes (n=540).

Data S3. qPLEX-RIME results for PRC1.3 interactions on chromatin. Summary of results for the qPLEX-RIME experiment for all proteins uniquely detected in the experiment (n=787), accompanied by their normalised protein intensities in each condition. Six pairwise comparisons of statistical significance between the four conditions are outlined in each sheet, including a log2 fold change and an adjusted *P*-value (Limma test with Benjamini-Hochberg correction).

Data S4. Further information related to the methods.
